# Interpolymer Complexation Between Cellulose Ethers, Poloxamers, and Polyacrylic Acid: Surface-Dependent Behavior

**DOI:** 10.3390/polym17101414

**Published:** 2025-05-21

**Authors:** Eldar Kopishev, Fatima Jafarova, Lyazat Tolymbekova, Gaini Seitenova, Ruslan Sаfarov

**Affiliations:** 1 Department of Chemistry, Faculty of Natural Sciences, L.N. Gumilyov Eurasian National University, Astana 010000, Kazakhstan; kopishev_eye@enu.kz (E.K.); dzhafarova_fn@enu.kz (F.J.); tolymbekova_lb@enu.kz (L.T.); 2 Department of Chemistry and Chemical Technology, Kh. Dosmukhamedov Atyrau University, Studenchesky Ave. 1, Atyrau 060011, Kazakhstan

**Keywords:** interpolymer complexes, poly(acrylic acid), drug delivery, cellulose ether, layer-by-layer deposition

## Abstract

This study examines the surface-dependent formation of interpolymer complexes (IPCs) by the layer-by-layer (LBL) deposition method. The materials used in this analysis are poly(acrylic acid) (PAA) combined with cellulose ethers, namely methyl cellulose (MC), hydroxypropyl cellulose (HPC), and hydroxyethyl cellulose (HEC), and poloxamers PX188 and PX407. PMMA, PS, and glass surfaces have been used to study the influence of hydrophobicity and hydrophilicity on IPC growth and its properties. Through contact angle measurements, PMMA and PS were found to be hydrophobic and glass hydrophilic. It was revealed by gravimetric analysis that IPC films reveal the highest growth on PMMA substrates, followed by PS and glass. Both the molecular weight of HEC and the hydrophobicity of the surface considerably affected the growth. Hydrogen-bonded complexation was evident by means of FTIR spectroscopy, while changes in some characteristic absorption bands demonstrated the extent of interactions between polymers. Scanning electron microscopy showed that variations in the microstructure of surfaces occur; PAA-MC and poloxamer complex layers were well organized on hydrophobic substrates. Thus, the experimental results showed surface properties, especially hydrophobicity, to be important for IPC growth and structure. These findings contribute to the understanding of IPC behavior on different substrates, thus giving insights into applications in drug delivery, coatings, and functional films.

## 1. Introduction

Interpolymer complexes (IPCs) represent a significant area of research [1,2,3,4] in polymer science due to their ability to form stable, functional materials through non-covalent interactions. These complexes, especially those based on cellulose ethers, have shown great promise in a variety of industries, such as food packaging, adhesives [5,6,7], coatings [8,9], and pharmaceuticals [10,11,12,13].

Interpolymer complexes arise from the interaction of macromolecules of different polymers with complementary chemical structures. Depending on the chemical makeup of the interacting polymers, these interactions, which are primarily non-covalent, include hydrophobic forces, electrostatic interactions, and hydrogen bonds. Both the chemical makeup of polymer chains and the aggregation of macromolecules, which results in higher-order structural topologies, influence the structural complexity of IPCs. Several physicochemical parameters, including temperature, polymer concentration, and solution pH, affect the formation of these complexes and regulate the type and strength of the interactions.

IPCs can be classified into four primary categories based on the nature of intermolecular interactions: Polyelectrolyte Complexes [14,15], Hydrogen-Bonded Complexes [16,17], Stereocomplexes [17,18], and Charge-Transfer Complexes [19,20].

The formation of IPCs can be achieved through several synthesis methods, each tailored to the specific properties required for the application: Solution Mixing [21,22], Matrix Polymerization [23,24], Interfacial Complexation [25,26], and Layer-by-Layer Deposition [27,28].

A comprehensive understanding of IPCs requires the use of several characterization techniques to analyze their structure, thermal properties, and functional behavior. The following methods are commonly employed: Titration [29,30], Viscometry and Turbidimetry [31,32], Potentiometry [33], Infrared (IR) Spectroscopy [34,35], Differential Scanning Calorimetry (DSC) [36,37,38], and Scanning Electron Microscopy (SEM) [39,40].

Interpolymer complexes can exhibit many positive properties in various areas of consumption, which explains their wide range of applications, such as in drug delivery systems [2,10,12], in coatings [8,9], as adhesives [5,6,7], and in food packaging.

Methylcellulose, propyl cellulose and hydroxyethyl cellulose, as well as poloxamers can form interpolymer complexes with polyacrylic acid based on hydrogen bonding.

**Figure 1:**
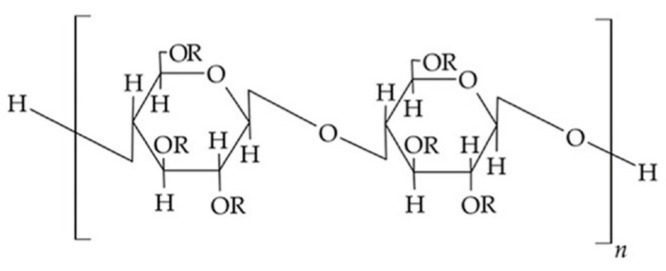
Chemical structure of cellulose esters.

Cellulose ethers (Figure 1) are derivatives of cellulose with the general chemical formula [C_6_H_7_O_2_(OH)_3−x_(OR)_x_]_n_, where X shows the number of hydroxyl groups that have been substituted in the cellulose macromolecule, while R represents an alkyl, acyl, or mineral acid residue. Methylcellulose, hydroxypropyl cellulose, and hydroxyethyl cellulose belong to cellulose ethers.

This article will examine the LBL deposition of interpolymer complexes of poly(acrylic acid) and cellulose ethers, as well as poloxamers on different surfaces, to study the intensity of their growth depending on surface properties.

**Figure 2:**
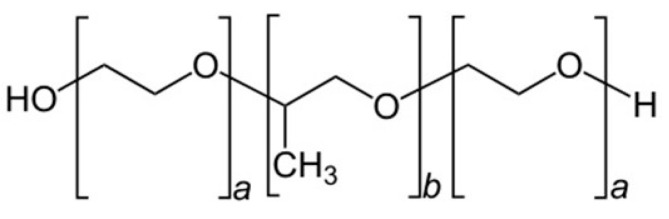
General chemical structure of poloxamer.

Poloxamers (Figure 2), also known by their trade name Pluronics, are a class of synthetic block copolymers that consist of a central hydrophobic block of poly(propylene oxide) (PPO) flanked by two hydrophilic blocks of poly(ethylene oxide) (PEO). Poloxamers are triblock copolymers with the general formula (PEO)_x_-(PPO)_y_-(PEO)_x_, where x and y represent the length of the ethylene oxide and propylene oxide chains (Figure 2).

The surface properties of different surfaces (microscope glass, polymethyl methacrylate, and polystyrene) significantly influence the formation and growth of interpolymer complexes (IPCs) between cellulose derivatives (methylcellulose, hydroxypropyl cellulose, and hydroxyethyl cellulose), poloxamers, and polyacrylic acid. Variations in surface properties such as hydrophilicity or roughness are expected to affect the molecular interactions and morphology of IPCs, leading to distinct structural characteristics based on the specific surface utilized for complexation, which can be quantitatively analysed through gravimetric methods, FTIR spectroscopy, and microphotography.

## 2. Materials and Methods

Poly(methyl methacrylate) (PMMA) slides and Polystyrene Microscope Slides (PSs) were purchased from Signimpress (Astana, Kazakhstan) and Super Premium Microscope Slides (76 mm × 26 mm) were purchased from VWR™ (West Chester, PA, USA).

Poly(acrylic acid) with Mv 450 kDa (PAA) and CAS Registry Number 9003-01-4; Methylcellulose (Methocel 60 HG, Mv 93 kDa, degree of substation 1.6, and molar substitution 2.0 mol per mol cellulose) (MC) with 28–30% methoxyl content and CAS Registry Number 9004-67-5; Hydroxypropyl cellulose with Mv 80 kDa (HPC) and CAS Registry Number 9004-64-2; 2-Hydroxyethyl cellulose with Mv 90 kDa, degree of substation 1.0, molar substitution 2.5 mol per mol cellulose (HEC90), and CAS Registry Number 9004-62-0; 2-Hydroxyethyl cellulose with Mv 250 kDa, degree of substitution 1.0, molar substitution 2.5 mol per mol cellulose (HEC250), and CAS Registry Number 9004-62-0; and 2-Hydroxyethyl cellulose Mv 720 kDa, with degree of substation 1.0, molar substitution 2.5 mol per mol cellulose (HEC720), and CAS Registry Number 9004-62-0 were purchased from Sigma-Aldrich (Burlington, MA, USA).

Lutrol F68 (PX188) and Kolliphor P407 (PX407) were purchased from BASF SE (Ludwigshafen, Germany).

All other chemicals, including methanol, 1 M hydrochloric acid (HCl), and concentrated sulfuric acid (H_2_SO_4_) were purchased from Sigma-Aldrich (West Chester, PA, USA).

Equipment used during the experiment:

AY220 Analytical Scales and an IRTracer-100 FTIR Spectrophotometer were purchased from SHIMADZU (Kyoto, Japan).

A Lab Disc White Magnetic Stirrer IKA C-MAG HS7 was purchased from IKA™ (Darmstadt, Germany).

A Seven Easy S20 pH Meter was purchased from Mettler Toledo (Greifensee, Switzerland).

During the experiment, prepared poly(methyl methacrylate), polystyrene, and microscope glass slides were used.

Impurity-free surfaces are necessary for effective and dependable polymer deposition on a solid planar glass surface. Free hydroxyl groups on the surface can also be released to enhance the deposition process. The method developed by Cras et al. [41,42,43] was used for surface preparation.

In total, 30 samples of each surface were immersed for 30 min in a 1:1 mixture of methanol/HCl (1 M), then rinsed in ultrapure H_2_O and dried at room temperature. Slides were then placed in concentrated H_2_SO_4_ for 30 min, then rinsed in ultrapure H_2_O and dried at room temperature. Following a 5 min immersion in a 1:5 HCl (1 M)/H_2_O combination at 80 °C, the slides were rinsed with water and allowed to dry. A desiccator was used to store the prepared slides for a maximum of 48 h before use.

The influence of the surface functional groups can be explained by measuring and calculating the contact angle of a sessile drop. For this purpose, water contact angle measurements of pure samples of PMMA, PS, and glass were carried out on Contact Angle Testing Equipment HD-U805 (Guangdong Haida Equipment Co., Ltd, Dongguan, China). Contact angle was measured at five different sample positions using a digital microscope with 1000× magnification by the static drop method at room temperature. The drop volume was 15 microliters.

A drop of water with a surface forms a contact angle, which depends on the roughness of the surface. Complete wettability is achieved when the contact angle tends to 0° and the liquid spreads over the surface. Weak wettability, on the contrary, is achieved when the contact angle tends to 180° and the liquid forms drops. It is generally accepted that a surface is hydrophilic if the contact angle is less than 90°, and if the contact angle is greater than 90° then it is hydrophobic.

**Figure 3:**
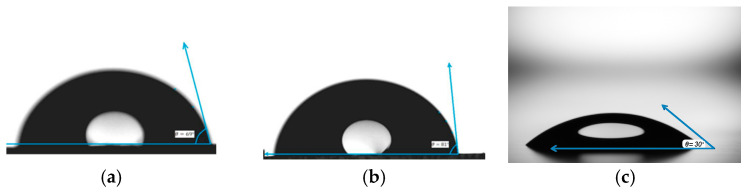
Representative contact angle measurements of water droplets on polymer and glass surfaces: (**a**) polymethyl methacrylate (PMMA), (**b**) polystyrene (PS), and (**c**) glass microscope slide. The contact angle (θ) indicates the hydrophobicity or hydrophilicity of each surface, with PMMA and PS exhibiting higher contact angles (θ = 69° and 81°, respectively), characteristic of hydrophobic behavior, while glass shows a significantly lower contact angle (θ = 30°), reflecting its hydrophilic nature. These measurements inform surface wettability and potential IPC film formation efficiency.

As shown in Figure 3, the contact angle of the liquid on polymethyl methacrylate is on average 69°, and on polystyrene 81°. The figure shows that the drop of liquid on the glass has spread comparatively over the surface, forming a contact angle of 30°. We can conclude that the surfaces of poly (methyl methacrylate) and polystyrene are hydrophobic, while the surface of glass is hydrophilic.

During preparation of polymer solutions for layer-by-layer deposition 2 g of Poly (acrylic acid) were dissolved in 1 L water; methylcellulose, hydroxypropyl cellulose, hydroxyethyl cellulose (Mv 90 kDa, 250 kDa, 720 kDa), and PX188 and PX407 weighing 1g each were separately dissolved in 0.5 L of deionized water and stirred on a magnetic stirrer for at least 8 h at room temperature until completely dissolved, obtaining 0.2% *w*/*v* solutions.

All polymer solutions were adjusted to pH 2.0 by adding small amounts of 1 M HCl and pH was monitored using a digital pH meter. An acidic water wash solution (pH 2) was also prepared by adding 1 M HCl (10 mL) to 1 L of deionized water.

All solutions, if not used immediately, were stored in the refrigerator until needed (for up to 3 days).

As is already known, two polymer solutions can be dissolved in distilled water and interpolymer films can be obtained by growing them on a flat surface using the layer-by-layer deposition method. This method grows very thin films (from 1 to 100 microns).

Pre-cleaned glass slides of each surface were chosen and weighed to create the multi-layered coating. It is assumed that the germination of the first polymer layer on the surface occurs due to hydrogen bonds of the carboxyl groups of polyacrylic acid with the surfaces.

250 mL 0.2% *w*/*v* poly(acrylic acid) solution, 250 mL 0.2% *w*/*v* methylcellulose, hydroxypropyl cellulose, hydroxyethyl cellulose (90, 250, and 720), PX (188 and 407) solutions and 500 mL of acidified water were poured into appropriate containers allowing slides to be submerged. There should be a 1 cm gap at the top to allow the slides to be handled.

To adhere the polymer coating to the surface, slides went through a dipping cycle. At first, the slides were placed in a polyacrylic acid solution for 15 min, then washed with acidified water for 30 s to get rid of unbound polymers. After this, the slides were immersed in polymer solutions for 15 min, followed by another 30 s washing in the acidified water. This cycle was repeated 10 times, resulting in 20 individual layers of polyacrylic acid and polymers, i.e., 10 layers of interpolymer complexes linked by hydrogen bonds.

Before analysis, multilayer coatings were air-dried at room temperature.

## 3. Results

### 3.1. Gravimetric Analysis

In this experiment, the surface densities of polymer layers on different surfaces were determined using gravimetric analysis. Analysis was conducted using a Shimadzu AY220 analytical balance with a readability of 0.1 mg.

Mass measurements were carried out before and after deposition of the interpolymer complexes on each surface. The obtained surfaces with IPC films were weighed on an analytical balance. The film area was measured in accordance with the immersion area. To avoid systematic and random errors, depending on the surface and the initial polymers, the same IPC films were obtained several times. The masses of the films were calculated by subtracting the initial surface mass from the final mass. The average mass of formed films was subsequently calculated. To calculate the specific weight of the formed films, the average mass of the formed films was divided by the surface area on which the film was formed. By calculating the specific weight of the samples, it is possible to determine the difference in the growth of films on surfaces, which will allow us to identify the optimal surface for further IPC growth, as well as draw conclusions depending on the nature of the surfaces. To identify accurate results, standard deviation and root mean square were also calculated.

**Figure 4:**
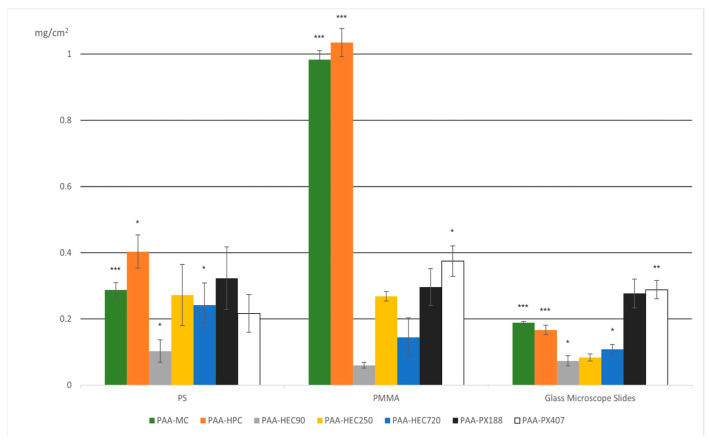
Surface density of interpolymer complex (IPC) films (mg/cm^2^) formed on various substrates (PS, PMMA, and glass microscope slides) using different polymer pairings. The bars represent the mean values of surface density for each IPC composition (e.g., PAA–MC, PAA–HPC, PAA–HEC variants, PAA–PX188, and PAA–PX407), with error bars indicating standard deviation. Statistical significance is indicated relative to a designated control (IPC film formed on glass), with *p*-values as follows: *** *p* < 0.001, ** *p* = 0.001–0.01, * *p* = 0.01–0.05, and none = not significant (*p* ≥ 0.05). These results highlight substrate-dependent differences in film formation efficiency, with PAA–MC and PAA–HEC250 exhibiting the highest deposition on PMMA.

The following diagram (Figure 4) shows the growth of IPC films based on the obtained mass results.

To further investigate whether there were statistically significant differences in the surface densities of interpolymer complexes on different surfaces, unpaired two-tailed Student’s *t*-tests [44] comparing each IPC–substrate pair to the designated control was used. Each data point represents the mean of three independent replicates (n = 3), with error bars indicating the standard deviation. Significance levels were assigned as follows: *** *p* < 0.001, ** *p* = 0.001–0.01, * *p* = 0.01–0.05, and none = not significant (*p* ≥ 0.05).

The null hypothesis (H_0_) is accepted if there are no significant differences between the surface densities of different IPCs on one surface.

During the *t*-tests, the surface densities of each interpolymer complex on various surfaces were evaluated. The findings indicated that for PAA-MC and PAA-HPC, significant differences in growth intensity were observed across all three surfaces. However, for PAA-HEC250 IPC, the growth differences across surfaces were insignificant. PAA-HEC90 and PAA-HEC720 exhibited significant differences in growth when comparing glass to PMMA, as well as glass to PS. Additionally, for PAA-PX407 differences in growth intensity were noted when comparing glass to PMMA and PMMA to PS.

### 3.2. Micrographs

**Figure 5:**
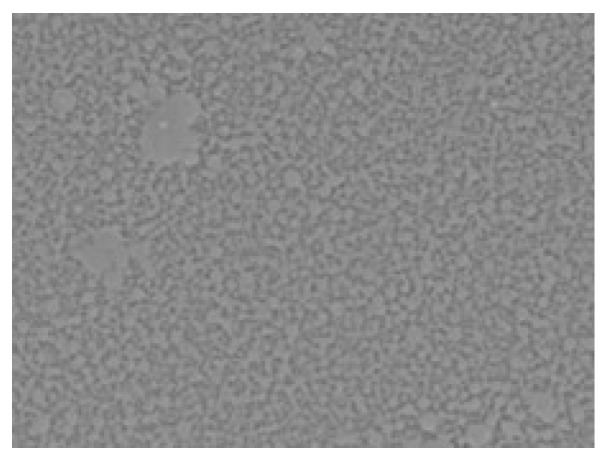
Scanning electron microscopy (SEM) image showing the surface morphology of the PAA–MC interpolymer complex formed on the PMMA substrate at 500× magnification. The image reveals a homogenous distribution of the IPC layer with granular microstructural features, indicating uniform deposition. Surface features suggest potential hydrogen bonding interactions between the polymers.

**Figure 6:**
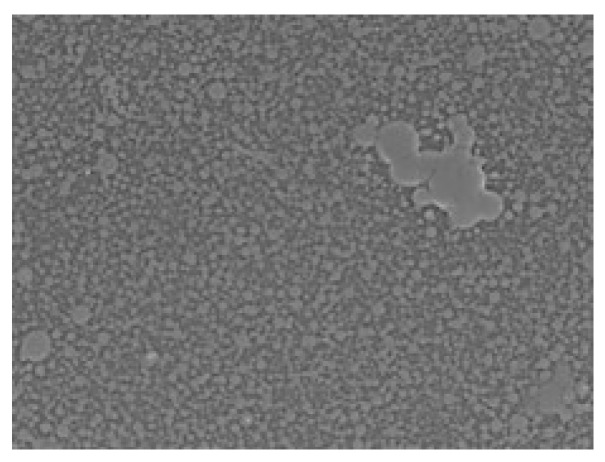
Scanning electron microscopy (SEM) image of the PAA–MC interpolymer complex formed on the surface of polystyrene (PS) at 500× magnification. The surface exhibits a densely packed microstructure with aggregated domains, suggesting partial phase separation or non-uniform interaction between the polymer components on the hydrophobic PS substrate.

**Figure 7:**
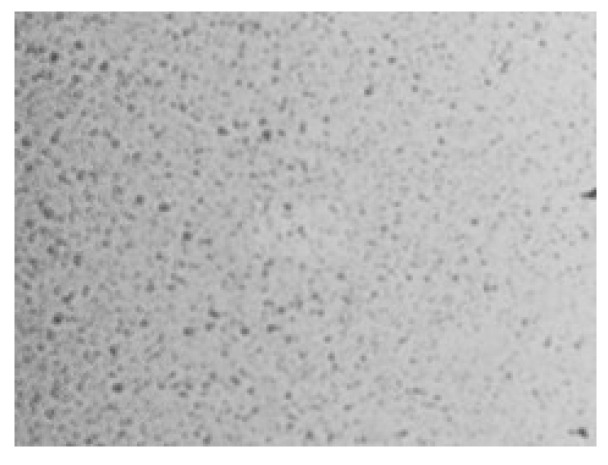
Scanning electron microscopy (SEM) image of the PAA–MC interpolymer complex deposited on glass at 500× magnification. Compared to polymer substrates, the surface shows a more heterogeneous and dispersed distribution of the IPC, with visible voids and irregular domains, likely due to weak polymer–substrate interactions on the highly hydrophilic glass surface.

To obtain a general picture of the microstructure of the obtained films, the samples were examined under a Hitachi TM3030 Tabletop Scanning Electron Microscope. Slides were examined before and after the application of the interpolymer complexes. These microscopy results allowed us to examine the microstructure of the IPC films with a magnification of 500 times. The results of the microstructure observation are shown in Figure 5, Figure 6 and Figure 7.

### 3.3. FTIR Spectroscopy

FTIR spectroscopy used an IRTracer-100 FTIR Spectrophotometer. This analysis was carried out to identify differences in peak intensity depending on the surfaces on which the polymer layers were applied.

Spectra were obtained within the wavenumber range of 4000–500 cm^−1^ at a resolution of 4 cm^−1^ for each sample. Each of the raw polymer samples were run as controls. The results of the IR spectra of the three graphs show a certain systematicity, which is quite understandable since the samples differ only in their surfaces.

## 4. Discussion

The study examined the surface densities of interpolymer complexes based on cellulose esters and poloxamers on different surfaces. For the experiment, prepared PMMA, PS, and microscope glass slides provided surfaces free of contaminants suitable for deposition. During the experiment, interpolymer complexes of PAA-MC, PAA-HPC, PAA-HEC90, PAA-HEC250, PAA-HEC720, PAA-PX188, and PAA-PX407 were obtained and studied.

As can be seen from the graph (Figure 4), the greatest film growth occurred on the surface of poly(methyl methacrylate), followed by polystyrene, and glass turned out to be the least suitable surface.

It is assumed that the high growth of the mass of IPC films on the PMMA surface is because the adhesion of polymers on the hydrophobic surface is the highest. Accordingly, the weak growth of films on the glass surface is due to its high wettability, due to which the deposited polymer layer was washed off during immersion in acidified distilled water.

In addition, on average, the growth of films based on MC and Poloxamers was higher than that of films based on HEC or HPC. The growth of IPC films based on PAA-HEC (720) is greater than that of PAA-HEC films with low molecular weight. This shows that it is easier to grow IPC films with the HEC (720) polymer solution than with the HEC (250) and HEC (90) polymer solutions.

The higher growth of IPC films using HEC (720) may be attributed to increased molecular weight, which likely enhances polymer chain entanglement and solution viscosity. These factors facilitate more stable and cohesive interpolymer interactions during layer-by-layer deposition. Previous studies suggest that higher molecular weights contribute to improved film integrity and adhesion, especially on hydrophobic surfaces.

This study does not include a systematic investigation into the kinetics of IPC formation, such as film thickness as a function of immersion time. This limitation restricts insight into the dynamic behavior of layer growth and bonding efficiency. We acknowledge this as a limitation of the current work and propose it as an important focus for future studies aiming to model deposition behavior quantitatively.

**Figure 8:**
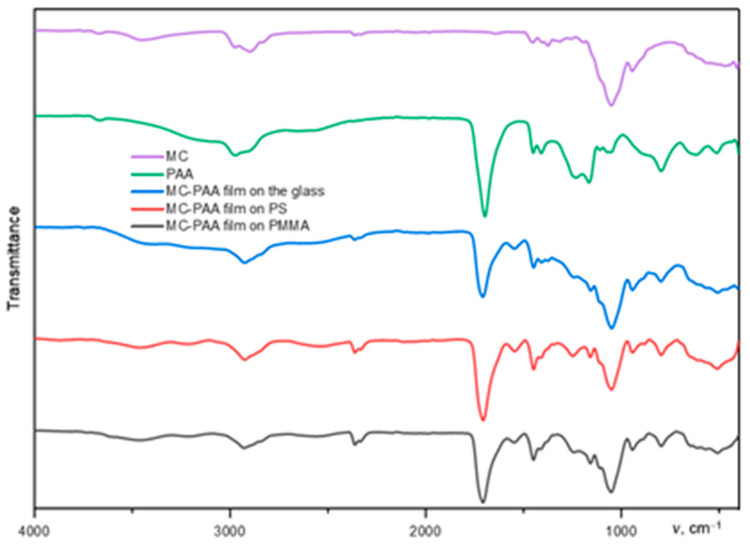
FT-IR spectrum of the deposited polymer film on a glass substrate. The key peaks correspond to the formation of hydrogen bonds according to the peaks in the region of 1700~1800 cm^−1^. This confirms the presence of polymer on the surface. FT-IR spectra of other films can be found in the Supplementary Materials.

FT-IR spectroscopy (Figure 8) can be used to analyze hydrogen-bonded complexation between PAA and cellulose esters, as well as poloxamers by tracking the shift of the characteristic absorbance band. Strong IR signals belonging to PAA, cellulose polymers, and poloxamers can be clearly observed in the IR spectra of PAA-HEC, PAA-MC, and PAA-PX films.

The FT-IR spectra revealed characteristic absorption bands at 3280 cm^−^^1^ (O–H stretching), 1640 cm^−^^1^ (C=O stretching), and 1050 cm^−^^1^ (C–O–C stretching). Upon IPC formation, the O–H stretching band shifted from 3280 cm^−^^1^ to 3255 cm^−^^1^, suggesting the formation of hydrogen bonds between the carboxylic groups of PAA and the hydroxyl groups of HEC. The shift in the C=O stretching band from 1640 to 1628 cm^−^^1^ further supports intermolecular interactions.

To quantitatively support the interpretation of hydrogen bonding, the characteristic wavenumber shifts for each IPC pair were measured and are summarized in Table 1. The O–H stretching band shifted from 3280 cm^−^^1^ to values ranging between 3255 and 3265 cm^−^^1^ depending on the polymer pair, while the C=O stretching band shifted from 1640 cm^−^^1^ to values between 1625 and 1632 cm^−^^1^. These shifts confirm the formation and strength of hydrogen bonding between PAA and the respective cellulose ethers or poloxamers.

The hydrogen bonding between CEs and PAA mainly happens between ether oxygen (or hydroxyl oxygen) of cellulose or poloxamer and COOH groups of PAA.

The results of the conducted IR spectroscopic studies show that the IR spectra of the obtained interpolymer complexes show prominent, broad, and well-defined peaks in the region of 1700~1800 cm^−1^, which can be attributed to the formation of hydrogen bonds in the initial polymers in the IPC films.

The hydrogen bond between the ether oxygen and COOH interrupts the hydrogen bond between the COOH groups. The shift in the spectrum can be used to describe the formation of hydrogen bonding between CEs or poloxamers and PAA. The more hydrogen bonds formed between PAA and initial polymers, the greater the shift in the C=O band compared to pure PAA in the IR spectra.

The micrographs (Figure 5, Figure 6 and Figure 7) show that on the surfaces of the slides where the films have grown there is clouding indicating successful deposition of the IPCs.

As can be seen in Figure 5, the film based on PAA-MC, PAA-PX188, and PAA-PX407 shows good layering on PMMA. Its structural organization consists of many individual sections that are linked together. The PAA-HEC films of different molecular weights form continuous, but loosely packed, macromolecular layers on the surface rather than sharply defined multilayers. It is assumed that these are macromolecules of the original polymers.

In Figure 6, the IPC films formed on the polystyrene surface showed similar growth patterns; however, the boundaries between phases were less defined. This difference may mean that on polystyrene, PAA-MC forms a semi-complex, but with low intensity, due to which clear boundaries of the formed PAA-MC are visible. In addition, PAA-PX188 layers are almost not visible on polystyrene. It can also be noticed individual sections of PAA-HEC (720) on the polystyrene surface, which differ from the microstructure of these polymers on the PMMA surface.

In Figure 7, the obtained microstructures of the IPC films on the glass surface correspond to the obtained data on mass growth intensity. Films based on PAA-MC and PAA-PX188 showed layering in the form of many clear circles. Films based on PAA-HEC with different molecular weights formed rare and small circles.

Based on the calculations of the contact angle, it can be assumed that the high growth of the mass of IPC films on the PMMA surface is because the adhesion of polymers on the hydrophobic surface is the highest. Accordingly, the weak growth of films on the glass surface may be due to its high wettability, due to which the deposited polymer layer was washed off during immersion in acidified distilled water.

The results show that the growth of poloxamers remained significant on each of the three surfaces, while cellulose ethers showed significant growth on the PMMA surface and growth on the glass surface was slow. Based on this, we can assume that such surface properties as its hydrophobicity and hydrophilicity have little effect on the formation of IPCs based on poloxamers, while they have a significant effect on the growth of IPCs based on glucose esters.

The difference in film growth behavior between poloxamer- and cellulose ether-based IPCs can be attributed to their molecular structures and interaction mechanisms. Cellulose ethers possess multiple hydroxyl groups, enabling extensive hydrogen bonding with PAA and resulting in more stable and cohesive IPC networks. In contrast, poloxamers, due to their amphiphilic block copolymer structure with hydrophilic PEO and hydrophobic PPO segments, engage in weaker, less directional hydrogen bonding. This molecular behavior accounts for the lower surface affinity and slower IPC film accumulation observed for poloxamer-based complexes, particularly on hydrophilic surfaces.

The observed differences between poloxamer- and cellulose ether-based IPCs are primarily due to their structural and interactive characteristics. Cellulose ethers possess multiple hydroxyl groups conducive to directional hydrogen bonding, leading to robust IPC film formation. Poloxamers, being amphiphilic triblock copolymers, engage more weakly due to their flexible, less polar PEO/PPO segments. This difference results in varied adhesion behavior and film morphology, depending on the substrate. Similar interpretations have been supported in the literature for amphiphilic polymer–surface interactions.

All the obtained results can be considered dependent on the number and strength of hydrogen bonds in the polymers. Methylcellulose (MC), hydroxypropyl cellulose (HPC), and hydroxyethyl cellulose (HEC), which are non-ionic cellulose ethers, are known to form interpolymer complexes via hydrogen bonding with polyacrylic acid (PAA). The formation of hydrogen bonds significantly influences not only the stability and association behavior of IPCs but also impacts their particle size, mass distribution, and surface morphology. Moreover, hydrogen bonding can modulate the electron density around functional groups, thereby affecting the electronic structure and potentially the interaction energy of the complexes. These molecular-level changes are reflected in the differences observed in IPC growth rates across different surfaces in our experiments.

This study showed the dependence of the formation of interpolymer complexes on the type and properties of the surface. The results obtained provide prospects for further research in this area. In the future, the influence of surface functionalization on its properties and, consequently, on the growth of IPC, as well as how functional groups on the surface affect the physicochemical properties of IPC can be investigated.

## 5. Conclusions

In has been shown that the chemical makeup of a surface is crucial for the efficiency of the deposition of interpolymer complexes using the layer-by-layer deposition technique because hydrogen-bonded polymer complexes are more readily deposited on surfaces that are inclined to hydrogen bonding. Depending on the initial polymers, the adhesion between a liquid and a surface has a positive effect on the growth of IPC films. A hydrophilic surface with high wettability leads to washing away polymers, which is why layering proceeds weakly. It is worth noting that surface properties affect not only the initial layer of polymer deposition but can also spread and affect the properties of the resulting multilayer film on the surface.

It was shown that interpolymer complexes based on PAA-MC, PAA-HEC (250), PAA-PX188, and PAA-PX407 are more prone to complex formation compared to interpolymer complexes based on PAA-HEC (90) and PAA-HEC (720).

The results show that the growth of poloxamers remained significant on each of the three surfaces, while cellulose ethers showed significant growth on the PMMA surface and growth on the glass surface was slow. Based on this, we can assume that such surface properties as its hydrophobicity and hydrophilicity have little effect on the formation of IPCs based on poloxamers, while they have a significant effect on the growth of IPCs based on glucose esters.

Of the three surfaces selected, poly(methyl methacrylate) turned out to be most optimal for layering polymer films.

## Figures and Tables

**Table 1:** Wavenumber shifts observed in FTIR spectra of interpolymer complexes.

IPC Pair	O–H Stretch (cm^−^^1^)	C=O Stretch (cm^−^^1^)
PAA–MC	3260	1632
PAA–HPC	3258	1629
PAA–HEC90	3257	1628
PAA–HEC250	3256	1626
PAA–HEC720	3255	1625
PAA–PX188	3265	1630
PAA–PX407	3263	1631

## Data Availability

The original contributions presented in this study are included in the article/Supplementary Materials. Further inquiries can be directed to the corresponding authors.

## Supplementary Materials

The following supporting information can be downloaded at https://www.mdpi.com/article/10.3390/polym17101414/s1, FT-IR spectra of other films can be found in the Supplementary Materials.

## References

[1] Keldibekova R., Suleimenova S., Nurgozhina G., Kopishev E. (2023). Interpolymer Complexes Based on Cellulose Ethers: Application. Polymers.

[2] Ramgonda P., Masareddy R.S., Patil A., Bolmal U. (2021). Development of Budesonide Oral Colon Specific Drug Delivery System Using Interpolymer Complexation Method. Indian J. Pharm. Educ. Res..

[3] Potaś J., Szymańska E., Basa A., Hafner A., Winnicka K. (2020). Tragacanth Gum/Chitosan Polyelectrolyte Complexes-Based Hydrogels Enriched with Xanthan Gum as Promising Materials for Buccal Application. Materials.

[4] Inagamov S.Y., Asrorov U.A., Xujanov E.B. (2023). Structure and Physico-Mechanical Properties of Polyelectrolyte Complexes Based on Sodium Carboxymethylcellulose Polysaccharide and Polyacrylamide. East Eur. J. Phys..

[5] Chen Y., Zhang L., Xu J., Xu S., Li Y., Sun R., Huang J., Peng J., Gong Z., Wang J. (2024). Development of a Hydroxypropyl Methyl Cellulose/Polyacrylic Acid Interpolymer Complex Formulated Buccal Mucosa Adhesive Film to Facilitate the Delivery of Insulin for Diabetes Treatment. Int. J. Biol. Macromol..

[6] Feldstein M.M., Dormidontova E.E., Khokhlov A.R. (2015). Pressure Sensitive Adhesives Based on Interpolymer Complexes. Prog. Polym. Sci..

[7] Boval’dinova K.A., Sherstneva N.E., Fel’dshtein M.M., Moskalets A.P., Khokhlov A.R. (2019). Pressure-Sensitive Adhesives with Tunable Tackiness. Polym. Sci. Ser. B.

[8] Khutoryanskiy V.V., Cascone M.G., Lazzeri L., Nurkeeva Z.S., Mun G.A., Mangazbaeva R.A. (2003). Phase Behaviour of Methylcellulose–Poly(Acrylic Acid) Blends and Preparation of Related Hydrophilic Films. Polym. Int..

[9] Iovescu A., Stîngă G., Maxim M.E., Gosecka M., Basinska T., Slomkowski S., Angelescu D., Petrescu S., Stănică N., Băran A. (2020). Chitosan-Polyglycidol Complexes to Coating Iron Oxide Particles for Dye Adsorption. Carbohydr. Polym..

[10] Boonlai W., Tantishaiyakul V., Hirun N., Sangfai T., Suknuntha K. (2018). Thermosensitive Poloxamer 407/Poly(Acrylic Acid) Hydrogels with Potential Application as Injectable Drug Delivery System. AAPS PharmSciTech.

[11] Raj H., Sharma A., Sharma S., Verma K.K., Chaudhary A. (2021). Mucoadhesive Microspheres: A Targeted Drug Delivery System. J. Drug Deliv. Ther..

[12] Kuperkar K., Atanase L., Bahadur A., Crivei I., Bahadur P. (2024). Degradable Polymeric Bio(Nano)Materials and Their Biomedical Applications: A Comprehensive Overview and Recent Updates. Polymers.

[13] Bianchera A., Bettini R. (2020). Polysaccharide Nanoparticles for Oral Controlled Drug Delivery: The Role of Drug–Polymer and Interpolymer Interactions. Expert Opin. Drug Deliv..

[14] Putro J.N., Lunardi V.B., Soetaredjo F.E., Yuliana M., Santoso S.P., Wenten I.G., Ismadji S. (2021). A Review of Gum Hydrocolloid Polyelectrolyte Complexes (PEC) for Biomedical Applications: Their Properties and Drug Delivery Studies. Processes.

[15] Bourganis V., Karamanidou T., Kammona O., Kiparissides C. (2017). Polyelectrolyte Complexes as Prospective Carriers for the Oral Delivery of Protein Therapeutics. Eur. J. Pharm. Biopharm..

[16] Gadwal I. (2020). A Brief Overview on Preparation of Self-Healing Polymers and Coatings via Hydrogen Bonding Interactions. Macromol.

[17] Munim S.A., Raza Z.A. (2019). Poly(Lactic Acid) Based Hydrogels: Formation, Characteristics and Biomedical Applications. J. Porous Mater..

[18] Schüttner S., Lu Y., Frey H., Coates G.W. (2024). Stereoregular Poly(Phenyl Glycidyl Ethers): In Situ Formation of a Polyether Stereocomplex from a Racemic Monomer Mixture. Angew. Chem. Int. Ed..

[19] Ma Y., Sun J., Shen J. (2007). Ion-Triggered Exfoliation of Layer-by-Layer Assembled Poly(Acrylic Acid)/Poly(Allylamine Hydrochloride) Films from Substrates: A Facile Way to Prepare Free-Standing Multilayer Films. Chem. Mater..

[20] El-Sayed M.Y., Refat M.S. (2014). The Intermolecular Charge-Transfer Complexes of the First Generation of Poly(Propylene Amine) Dendrimers with g and n Acceptors. Int. J. Electrochem. Sci..

[21] Pergushov D.V., Borisov O.V., Zezin A.B., Müller A.H.E. (2010). Interpolyelectrolyte Complexes Based on Polyionic Species of Branched Topology. Self Organized Nanostructures of Amphiphilic Block Copolymers I.

[22] Shovsky A., Varga I., Makuška R., Claesson P.M. (2009). Formation and Stability of Water-Soluble, Molecular Polyelectrolyte Complexes: Effects of Charge Density, Mixing Ratio, and Polyelectrolyte Concentration. Langmuir.

[23] Salimi H., Aryanasab F., Banazadeh A.R., Shabanian M., Seidi F. (2016). Designing Syntheses of Cellulose and Starch Derivatives with Basic or Cationic *N*-functions: Part I—Cellulose Derivatives. Polym. Adv. Technol..

[24] Papisov I.M., Kuzovleva O.E., Markov S.V., Litmanovich A.A. (1984). Chemical and Structural Modification of Polymers by Matrix Polymerization. Eur. Polym. J..

[25] Driver K., Baco S., Khutoryanskiy V.V. (2013). Hollow Capsules Formed in a Single Stage via Interfacial Hydrogen-Bonded Complexation of Methylcellulose with Poly(Acrylic Acid) and Tannic Acid. Eur. Polym. J..

[26] Fang S., Guan K., Mai Z., Zhou S., Song Q., Li Z., Xu P., Hu M., Chiao Y.-H., Zhang P. (2023). Complexation of Cellulose Nanocrystals and Amine Monomer for Improved Interfacial Polymerization of Nanofiltration Membrane. J. Memb. Sci..

[27] KHUTORYANSKIY V. (2007). Hydrogen-Bonded Interpolymer Complexes as Materials for Pharmaceutical Applications. Int. J. Pharm..

[28] Wang C., Pham D.A., Zhang H., Rabanel J., Hassanpour N., Banquy X. (2024). Layer-by-Layer Deposition of a Polycationic Bottlebrush Polymer with Hyaluronic Acid Reveals Unusual Assembly Mechanism and Selective Effect on Cell Adhesion and Fate. Adv. Funct. Mater..

[29] Bizley S.C., Williams A.C., Khutoryanskiy V.V. (2014). Thermodynamic and Kinetic Properties of Interpolymer Complexes Assessed by Isothermal Titration Calorimetry and Surface Plasmon Resonance. Soft Matter.

[30] Shestakova D.O., San’kova N.N., Parkhomchuk E.V. (2023). Conductometric and Potentiometric Titration of Carboxyl Groups in Polymer Microspheres. Polym. Sci. Ser. A.

[31] Morariu S., Avadanei M., Nita L.E. (2023). Effect of PH on the Poly(Acrylic Acid)/Poly(Vinyl Alcohol)/Lysozyme Complexes Formation. Molecules.

[32] Croitoru C., Roata I.C., Pascu A., Stanciu E.M. (2020). Diffusion and Controlled Release in Physically Crosslinked Poly (Vinyl Alcohol)/Iota-Carrageenan Hydrogel Blends. Polymers.

[33] Piela P., Mitzel J., Rosini S., Tokarz W., Valle F., Pilenga A., Malkow T., Tsotridis G. (2020). Looking Inside Polymer Electrolyte Membrane Fuel Cell Stack Using Tailored Electrochemical Methods. J. Electrochem. Energy Convers. Storage.

[34] Ruiz-Rubio L., Vilas J.L., Rodríguez M., León L.M. (2014). Thermal Behaviour of H-Bonded Interpolymer Complexes Based on Polymers with Acrylamide or Lactame Groups and Poly(Acrylic Acid): Influence of N-Alkyl and α-Methyl Substitutions. Polym. Degrad. Stab..

[35] Tomić S.L., Filipović J.M. (2004). Synthesis and Characterization of Complexes between Poly(Itaconic Acid) and Poly(Ethylene Glycol). Polym. Bull..

[36] Liew C.-W., Ramesh S., Arof A.K. (2015). Characterization of Ionic Liquid Added Poly(Vinyl Alcohol)-Based Proton Conducting Polymer Electrolytes and Electrochemical Studies on the Supercapacitors. Int. J. Hydrogen Energy.

[37] Drzeżdżon J., Jacewicz D., Sielicka A., Chmurzyński L. (2019). Characterization of Polymers Based on Differential Scanning Calorimetry Based Techniques. TrAC Trends Anal. Chem..

[38] Noskov A.V., Alekseeva O.V., Guseinov S.S. (2023). A Differential Scanning Calorimetry Study of Phase Transitions in Ethyl Cellulose/Bentonite Polymer Composites. Prot. Met. Phys. Chem. Surf..

[39] Slyusarenko N.V., Vasilyeva N.Y., Kazachenko A.S., Gerasimova M.A., Romanchenko A.S., Slyusareva E.A. (2020). Synthesis and Properties of Interpolymer Complexes Based on Chitosan and Sulfated Arabinogalactan. Polym. Sci. Ser. B.

[40] Farahani B.V., Shalbafan M. (2020). Synthesis, Controlled Release and Kinetic Studies of Polyacrylic Acid-Polyethylene Oxide/β-Cyclodextrin Nano-Interpolymer Complex with Naproxen. Orbital Electron. J. Chem..

[41] Cras J.J., Rowe-Taitt C.A., Nivens D.A., Ligler F.S. (1999). Comparison of Chemical Cleaning Methods of Glass in Preparation for Silanization. Biosens. Bioelectron..

[42] Leibauer B., Pop-Georgievski O., Sosa M.D., Dong Y., Tremel W., Butt H.-J., Steffen W. (2024). How Surface and Substrate Chemistry Affect Slide Electrification. J. Am. Chem. Soc..

[43] Pyo M., Jeong S., Kim J.H., Jeon M.J., Lee E.-J. (2024). Hydrophobicity and Membrane Distillation Performance of Glass Fiber Membranes Modified by Dip Coating of Pure PDMS. J. Environ. Chem. Eng..

[44] Anders K. (2017). Resolution of Students T-Tests, ANOVA and Analysis of Variance Components from Intermediary Data. Biochem. Med..

