# Supplementary figures and images for: Interpolymer Complexation Between Cellulose Ethers, Poloxamers, and Polyacrylic Acid: Surface-Dependent Behavior
Source: Polymers (Basel). 2025 May 21;17(10):1414. doi: 10.3390/polym17101414 (PMC12114889; doi:10.3390/polym17101414)

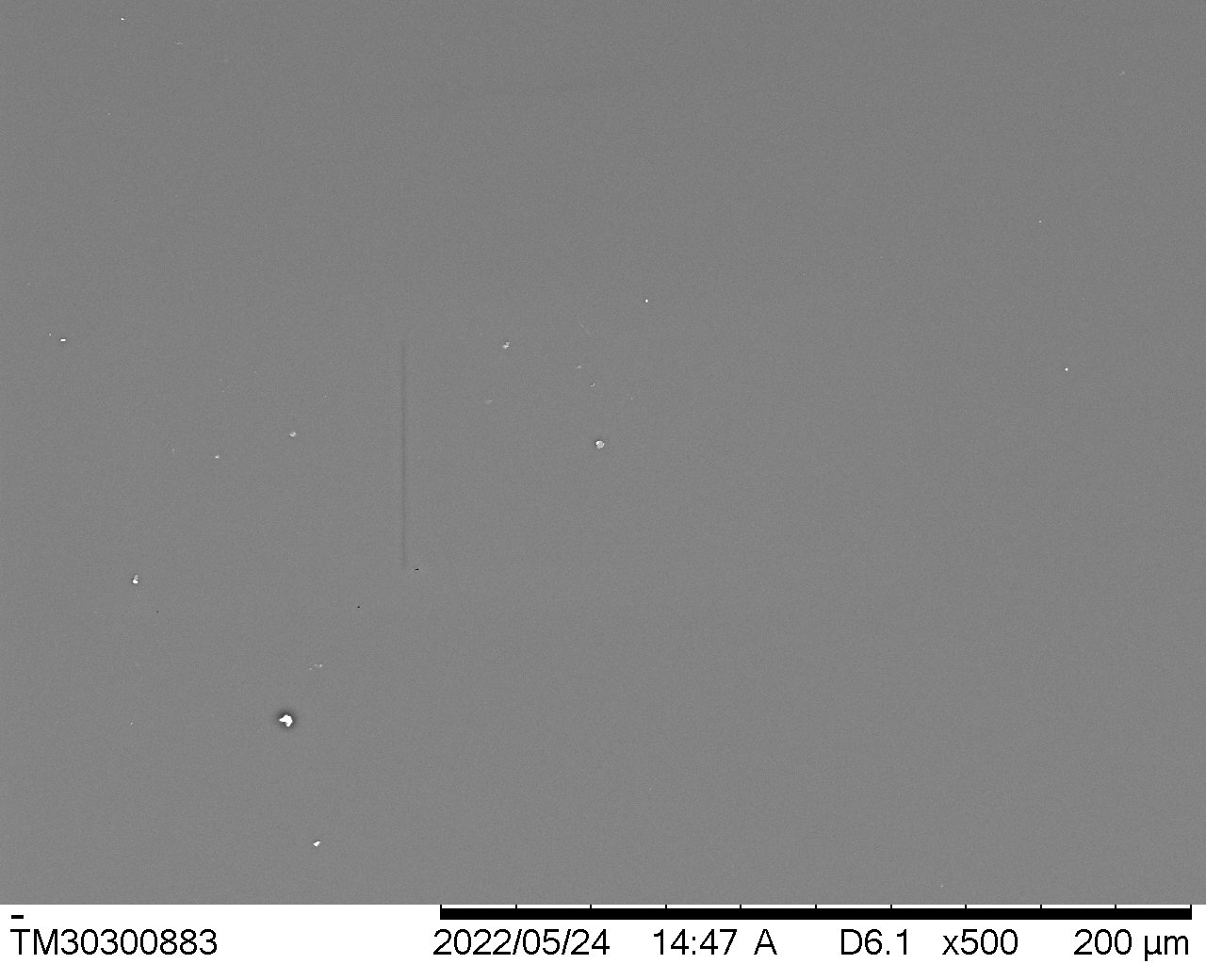

Supplement: Supplementary file 1 [file polymers-17-01414-s001.zip › isx_01_(x500).jpg]

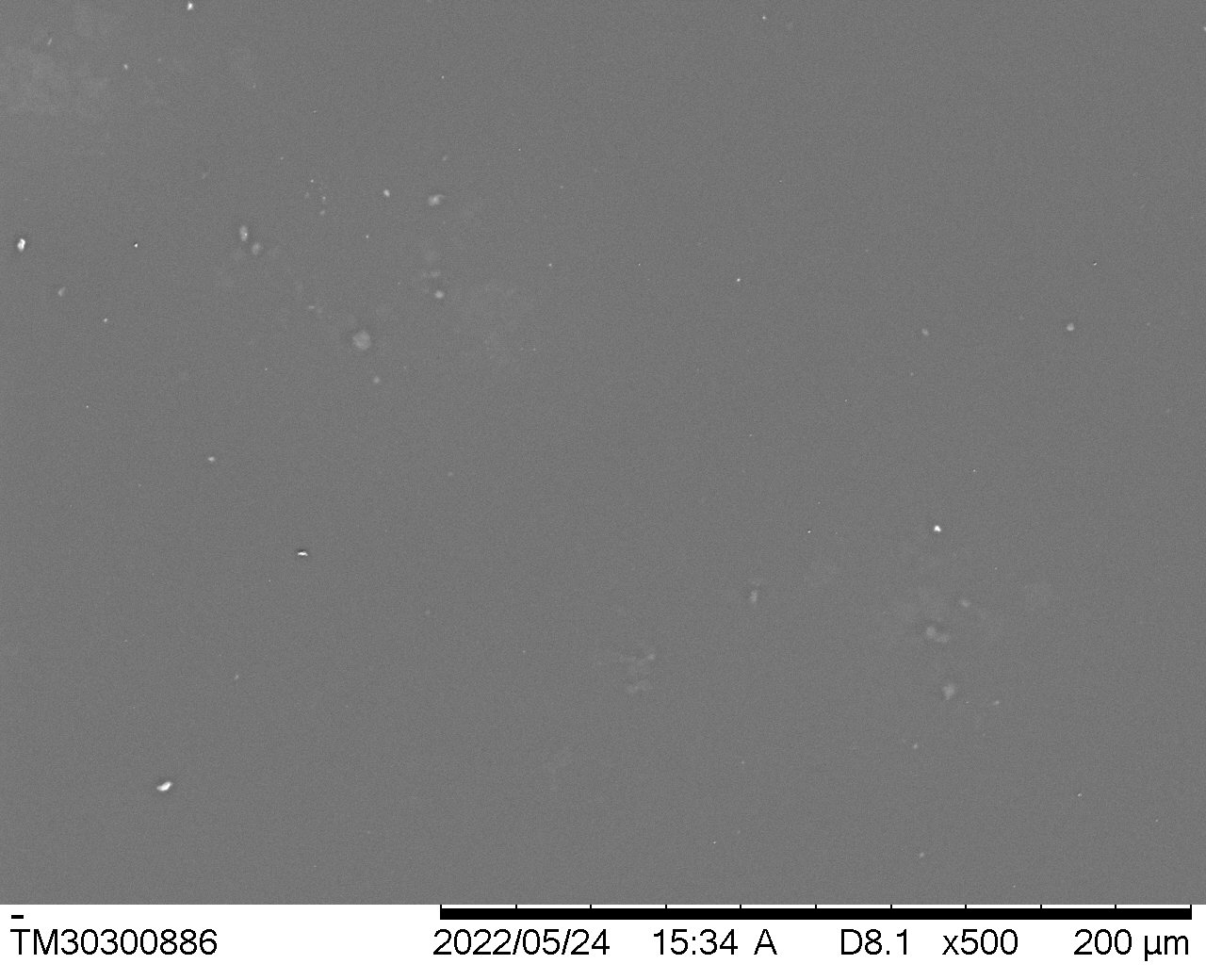

Supplement: Supplementary file 1 [file polymers-17-01414-s001.zip › PMMA_90_02_(x500).jpg]

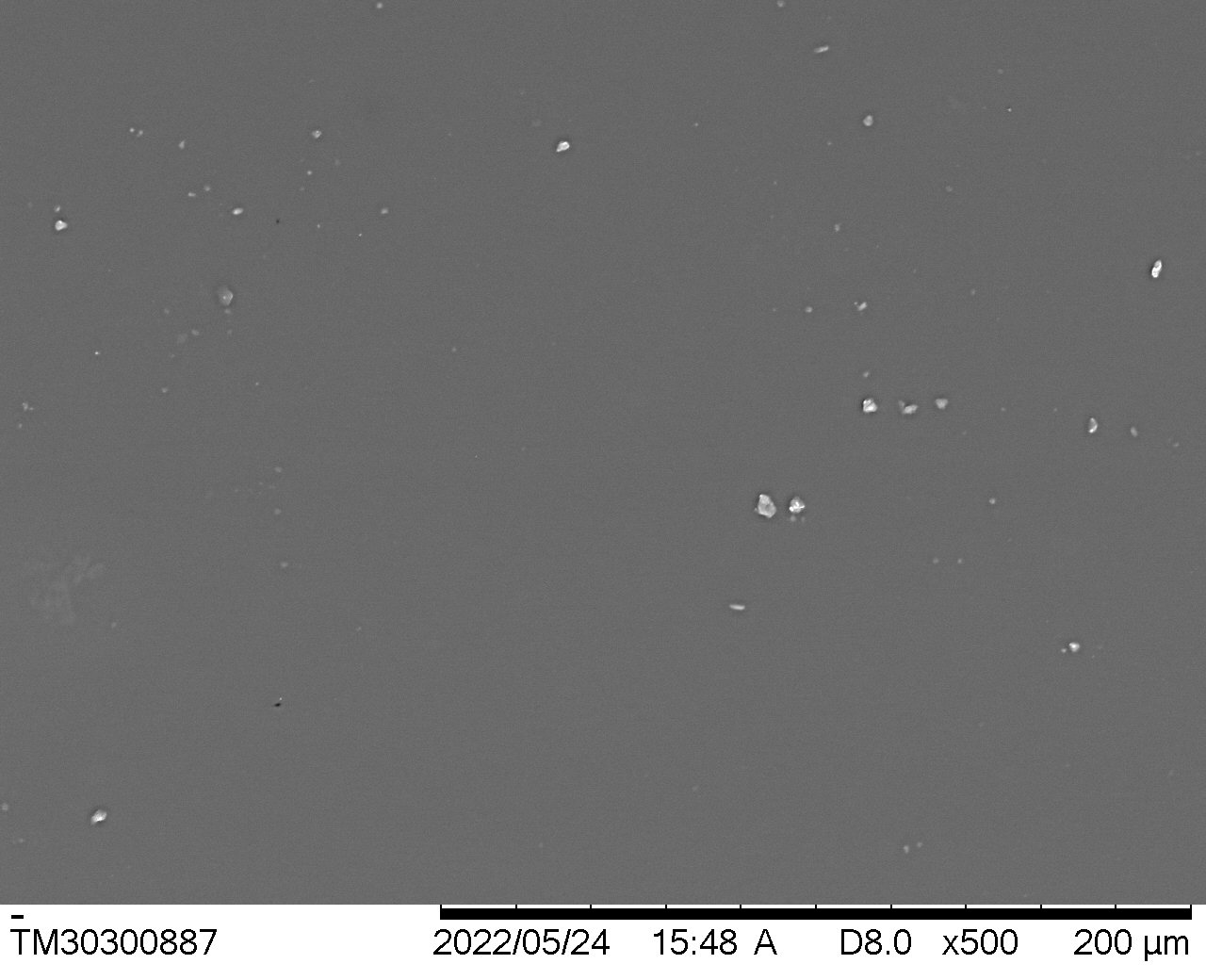

Supplement: Supplementary file 1 [file polymers-17-01414-s001.zip › PMMA_250_01_(x500).jpg]

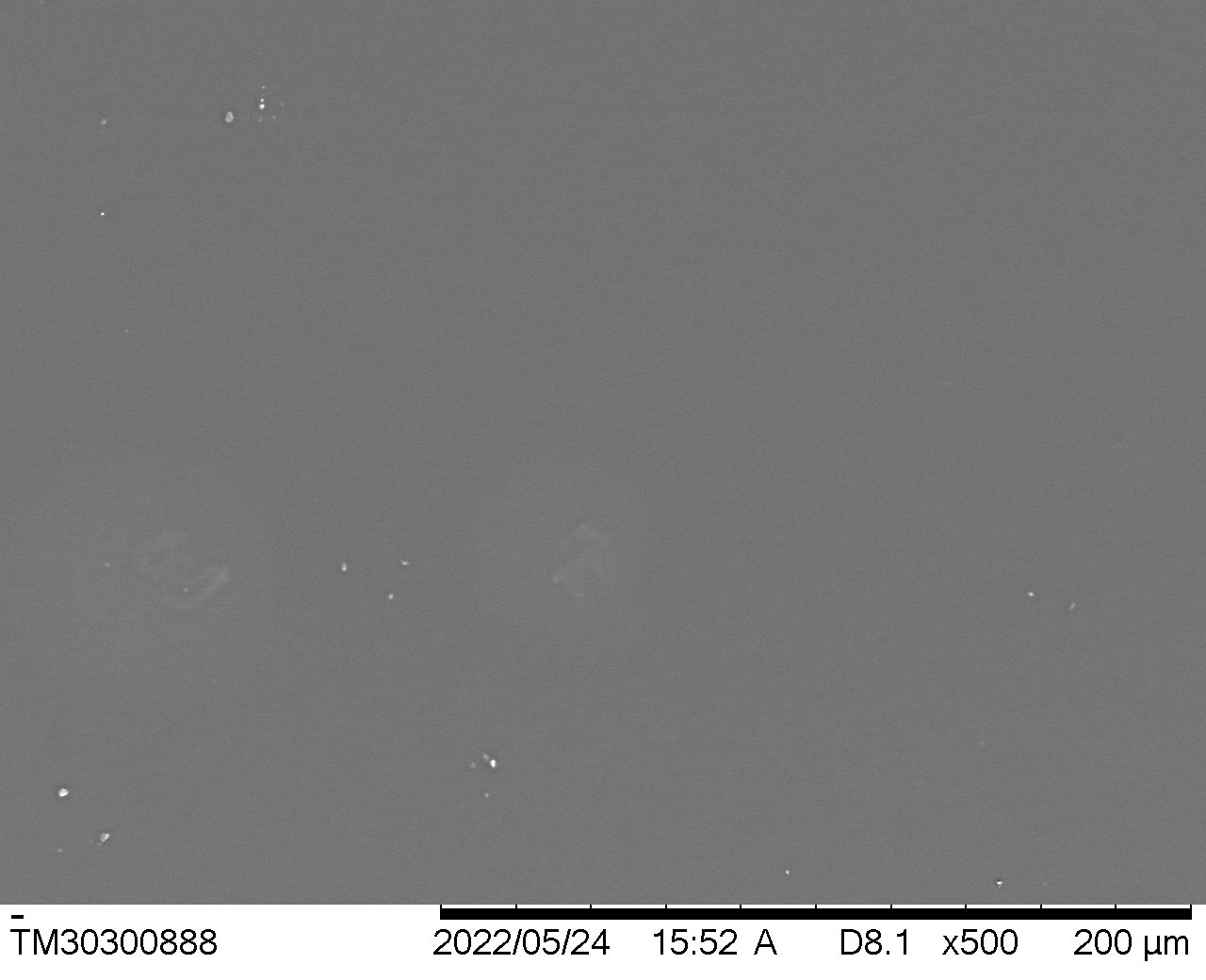

Supplement: Supplementary file 1 [file polymers-17-01414-s001.zip › PMMA_250_02_(x500).jpg]

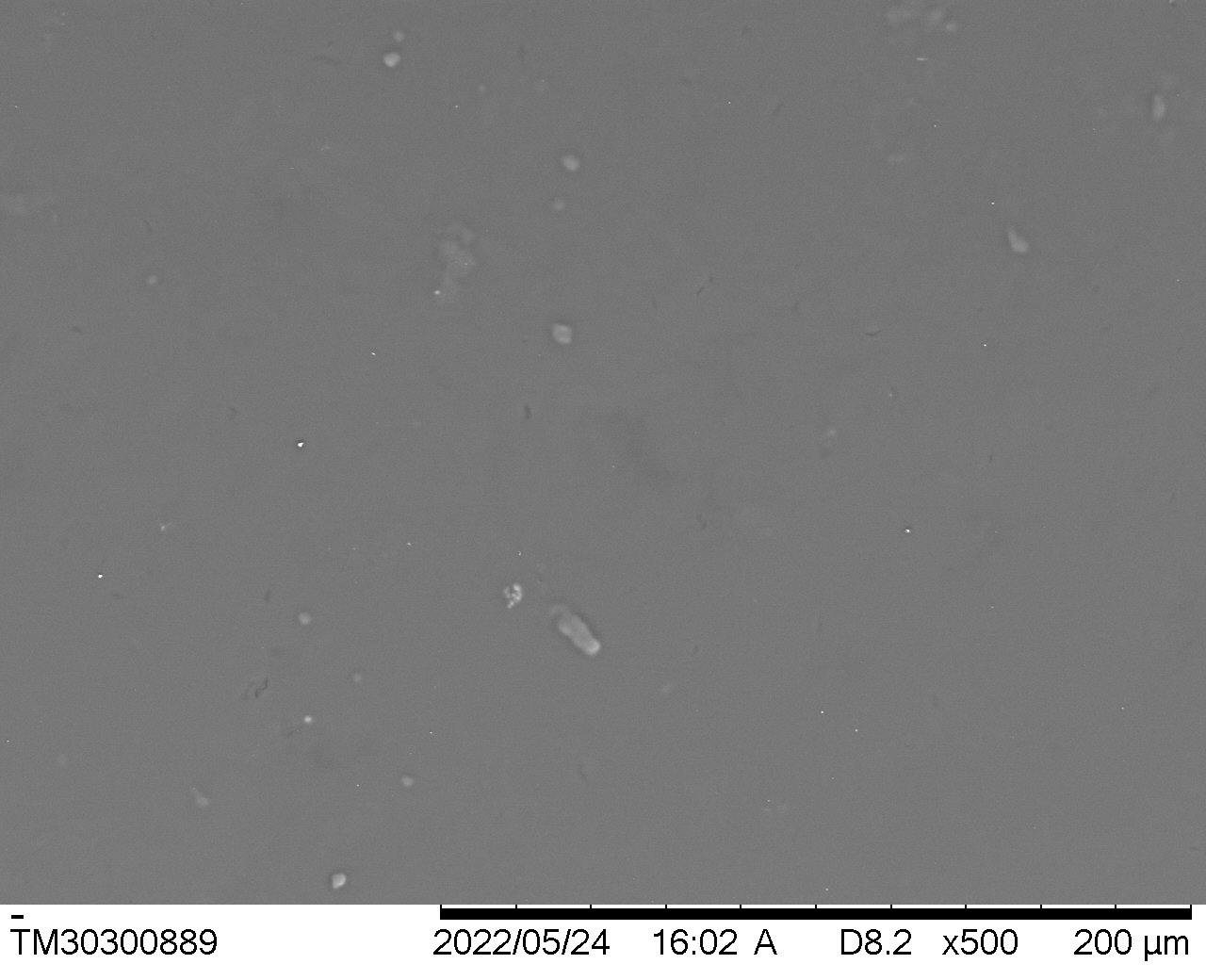

Supplement: Supplementary file 1 [file polymers-17-01414-s001.zip › PMMA_720_01_(x500).jpg]

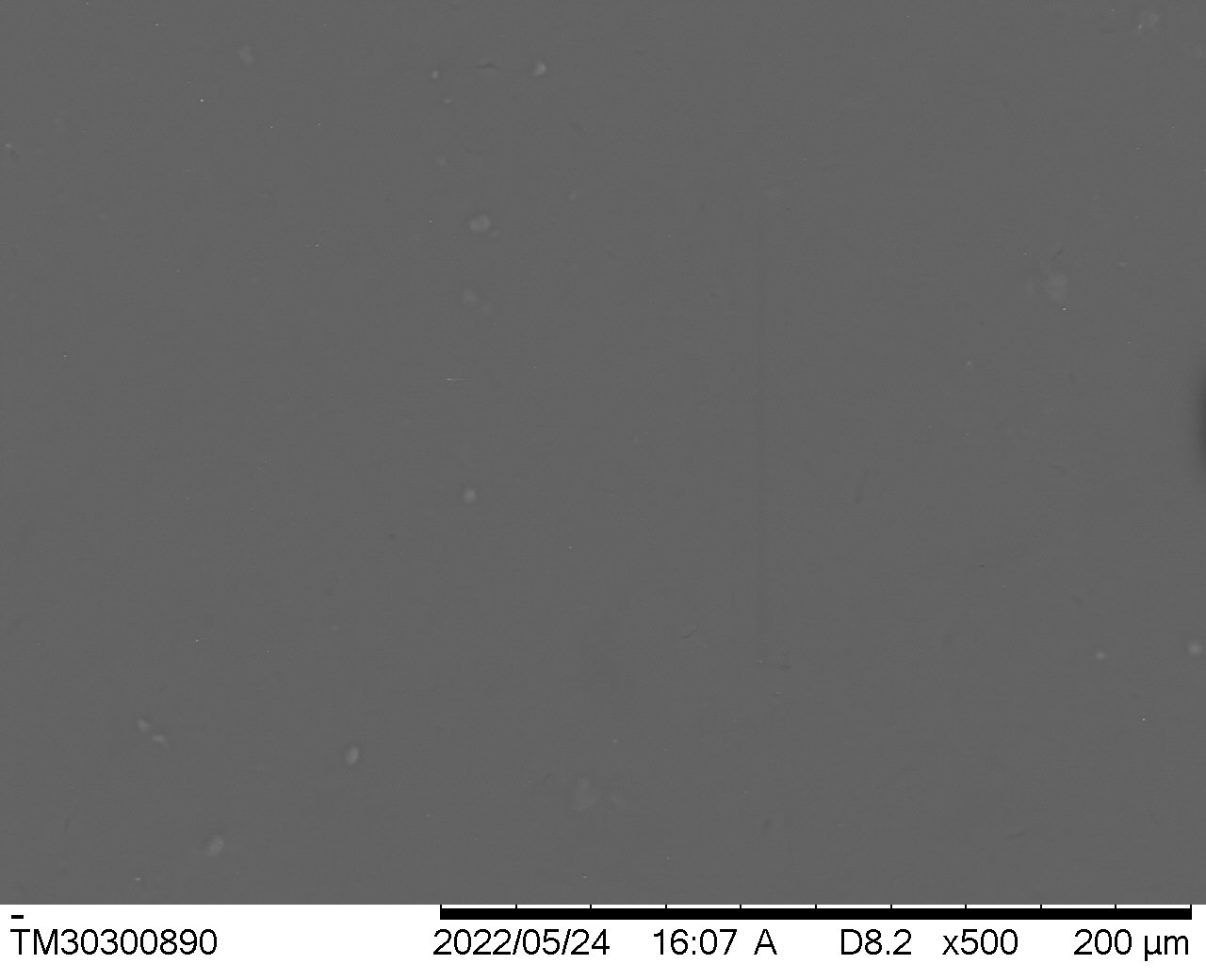

Supplement: Supplementary file 1 [file polymers-17-01414-s001.zip › PMMA_720_02_(x500).jpg]

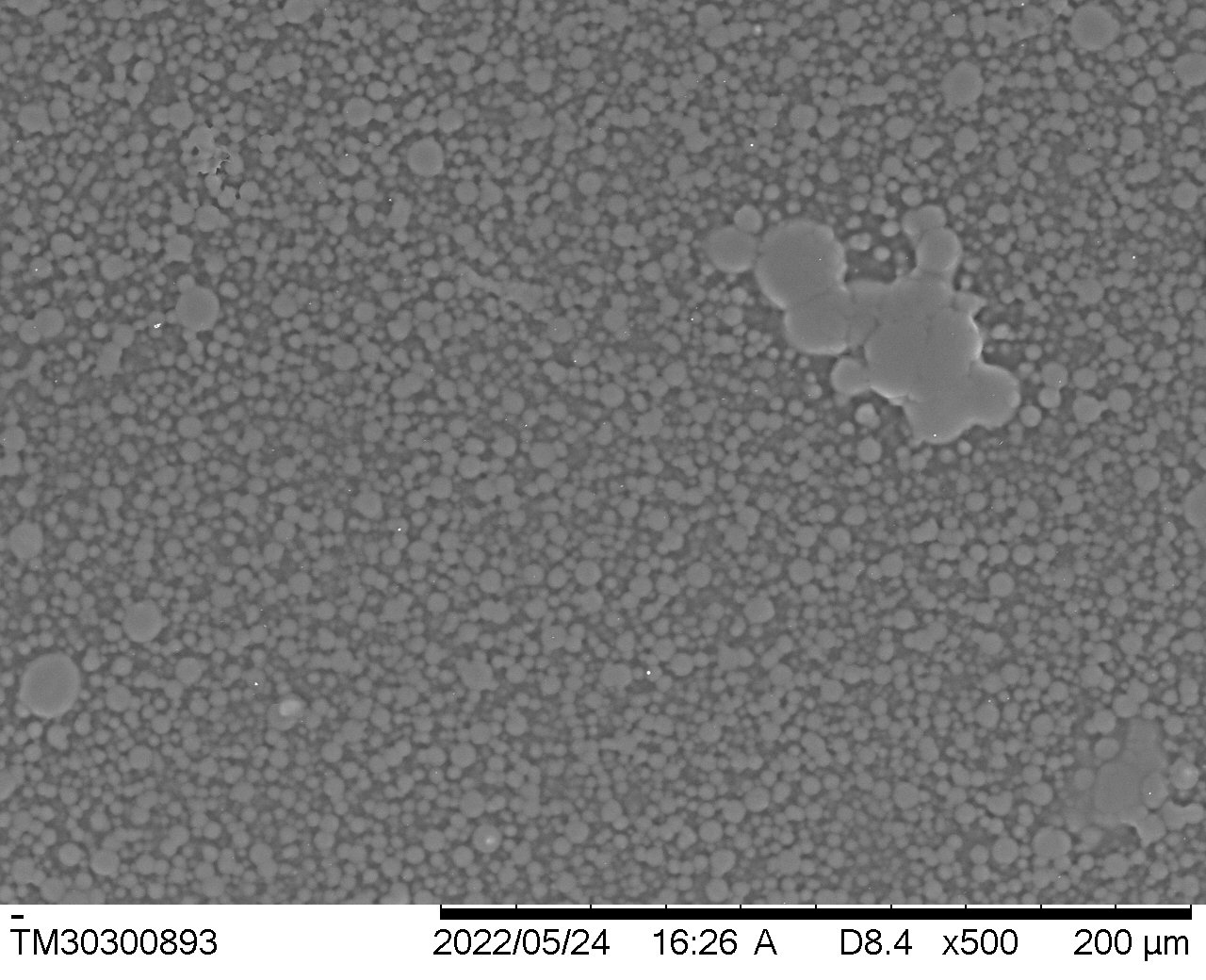

Supplement: Supplementary file 1 [file polymers-17-01414-s001.zip › Pc_60_01_(x500).jpg]

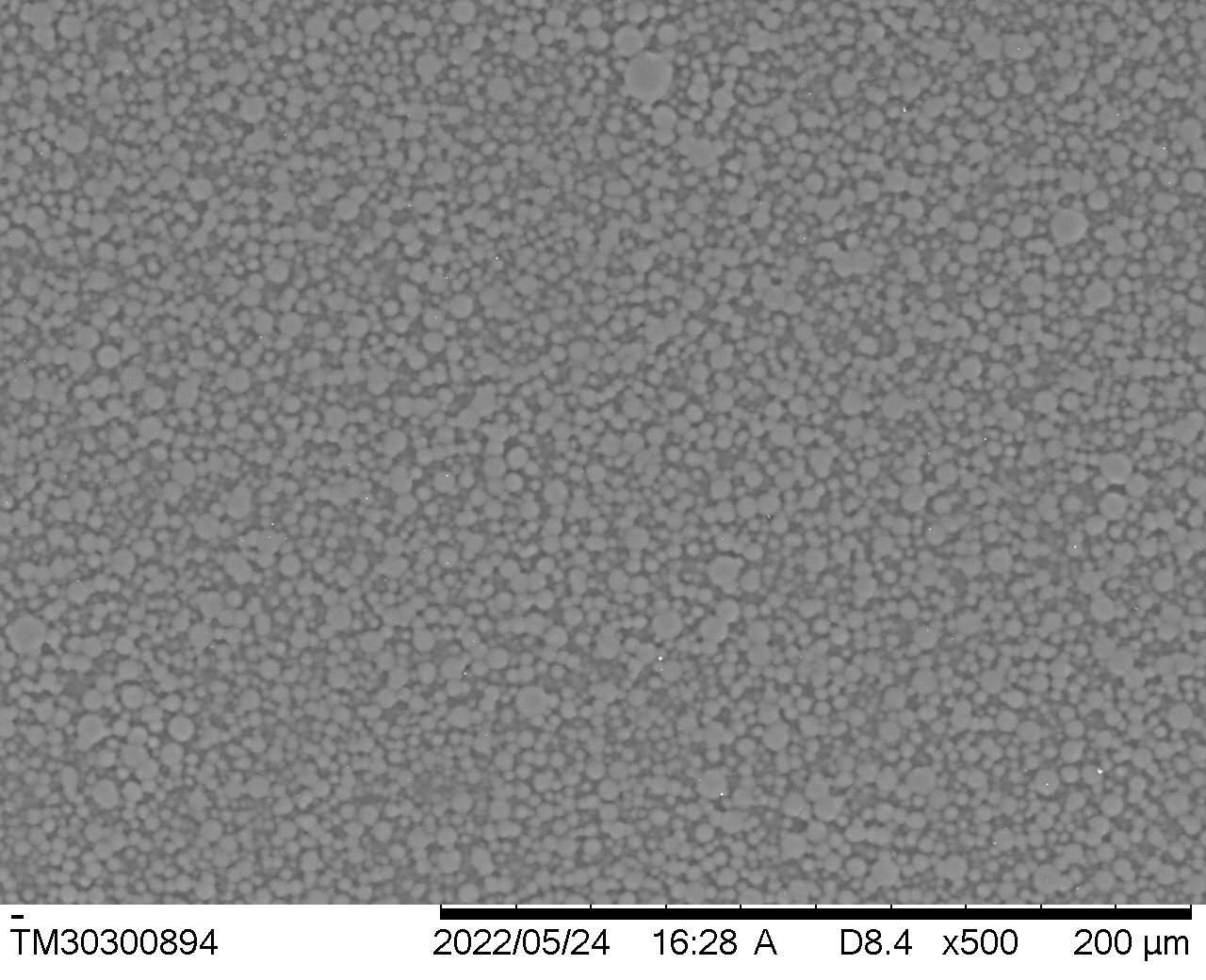

Supplement: Supplementary file 1 [file polymers-17-01414-s001.zip › Pc_60_02_(x500).jpg]

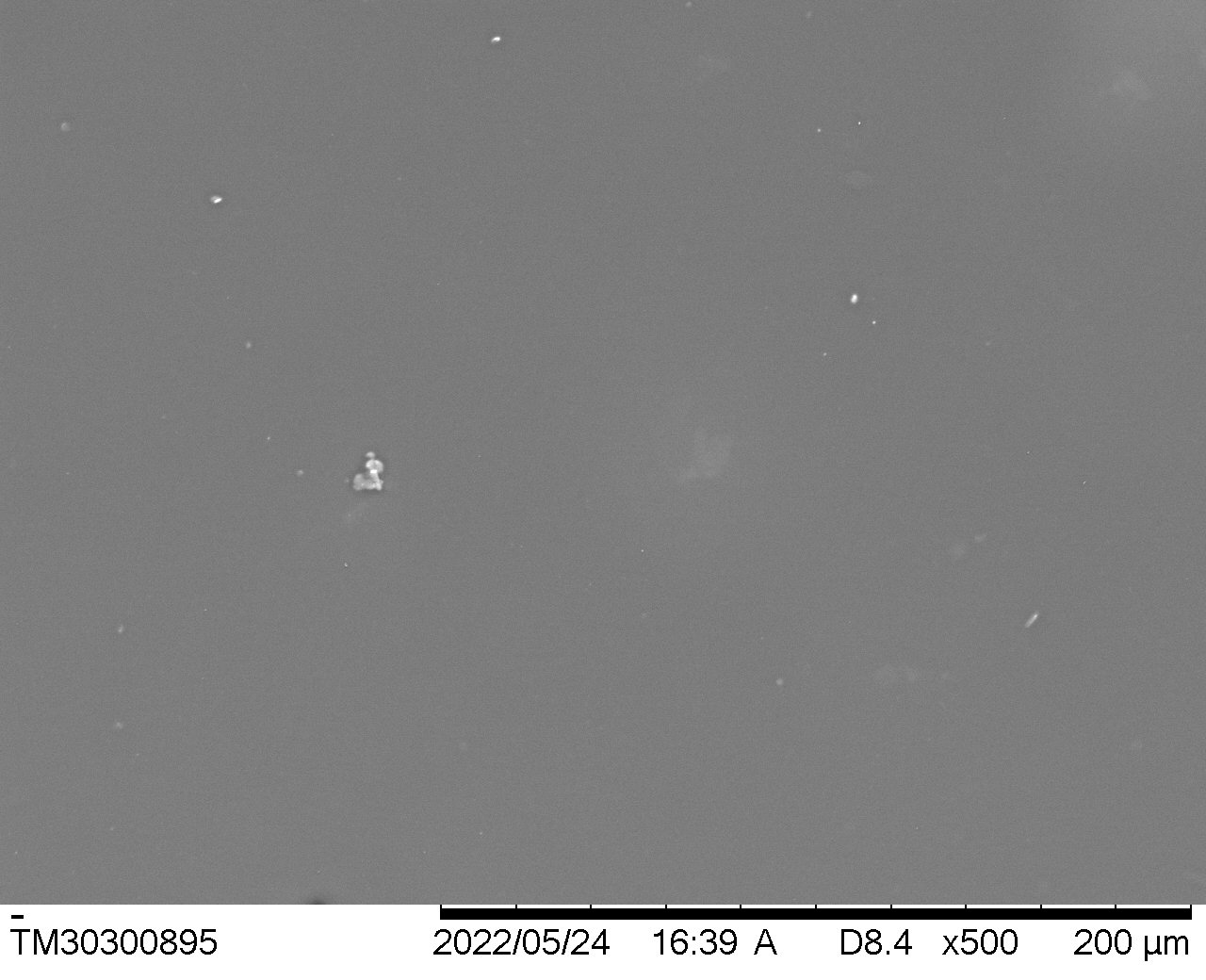

Supplement: Supplementary file 1 [file polymers-17-01414-s001.zip › Pc_90_01_(x500).jpg]

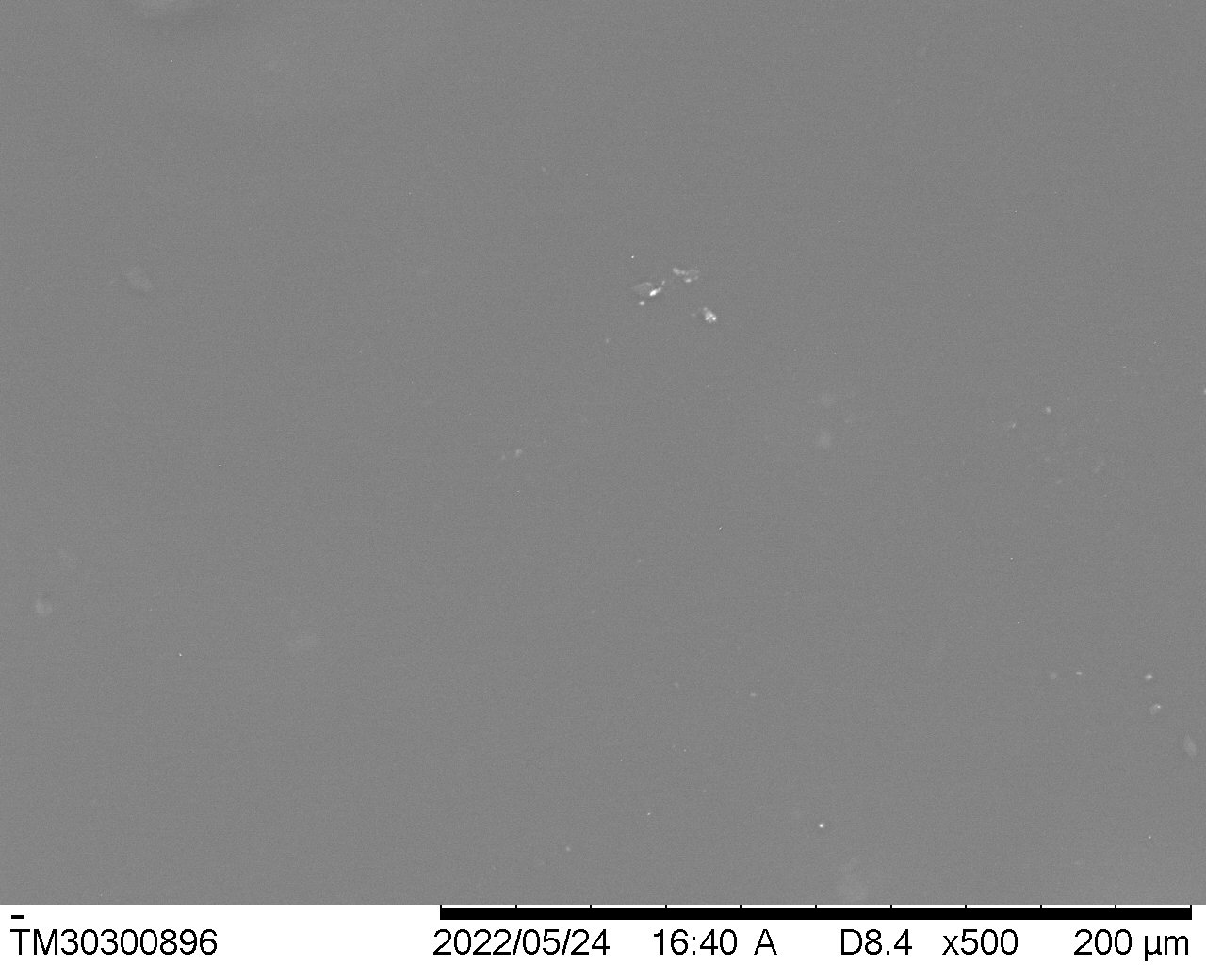

Supplement: Supplementary file 1 [file polymers-17-01414-s001.zip › Pc_90_02_(x500).jpg]

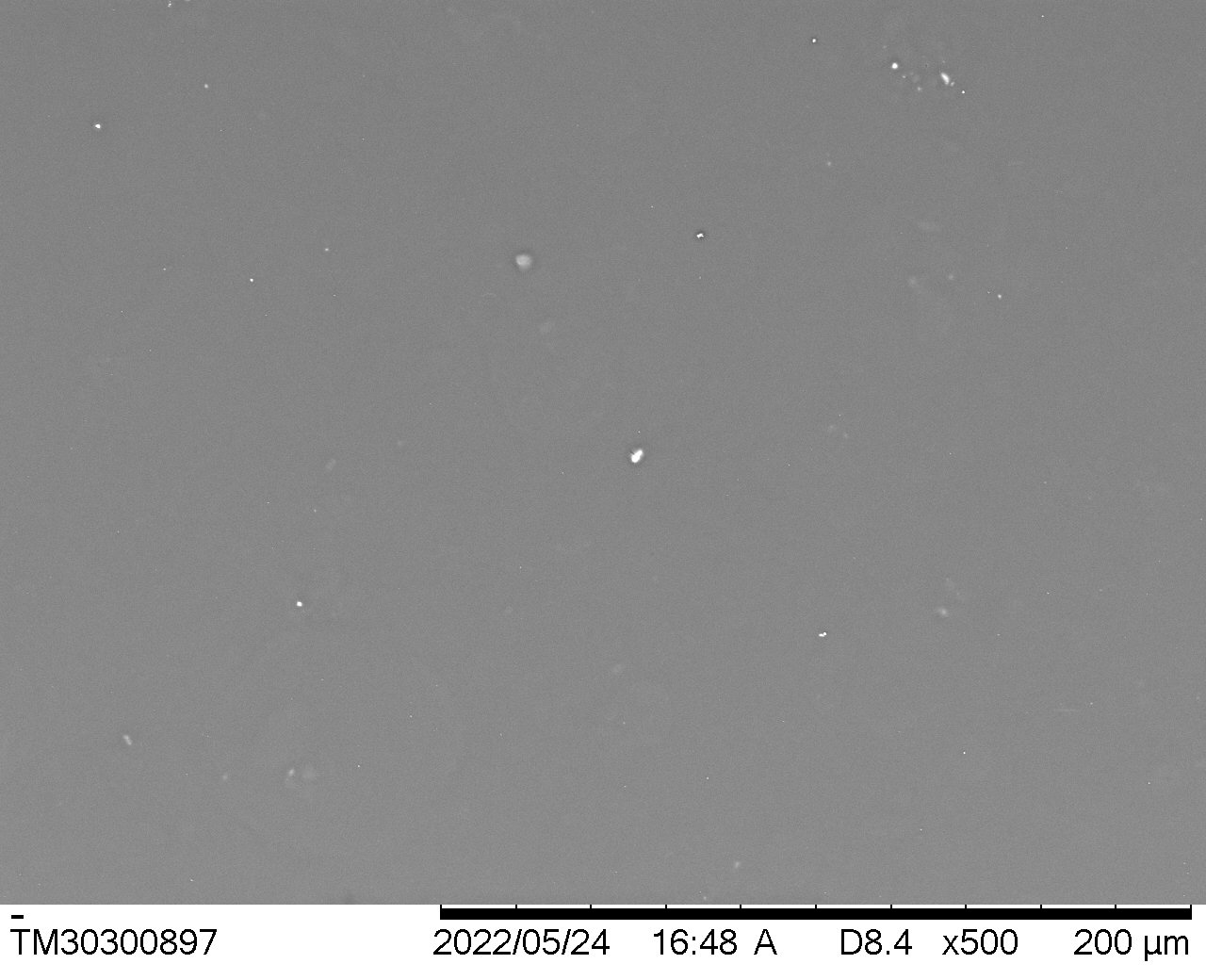

Supplement: Supplementary file 1 [file polymers-17-01414-s001.zip › Pc_250_01_(x500).jpg]

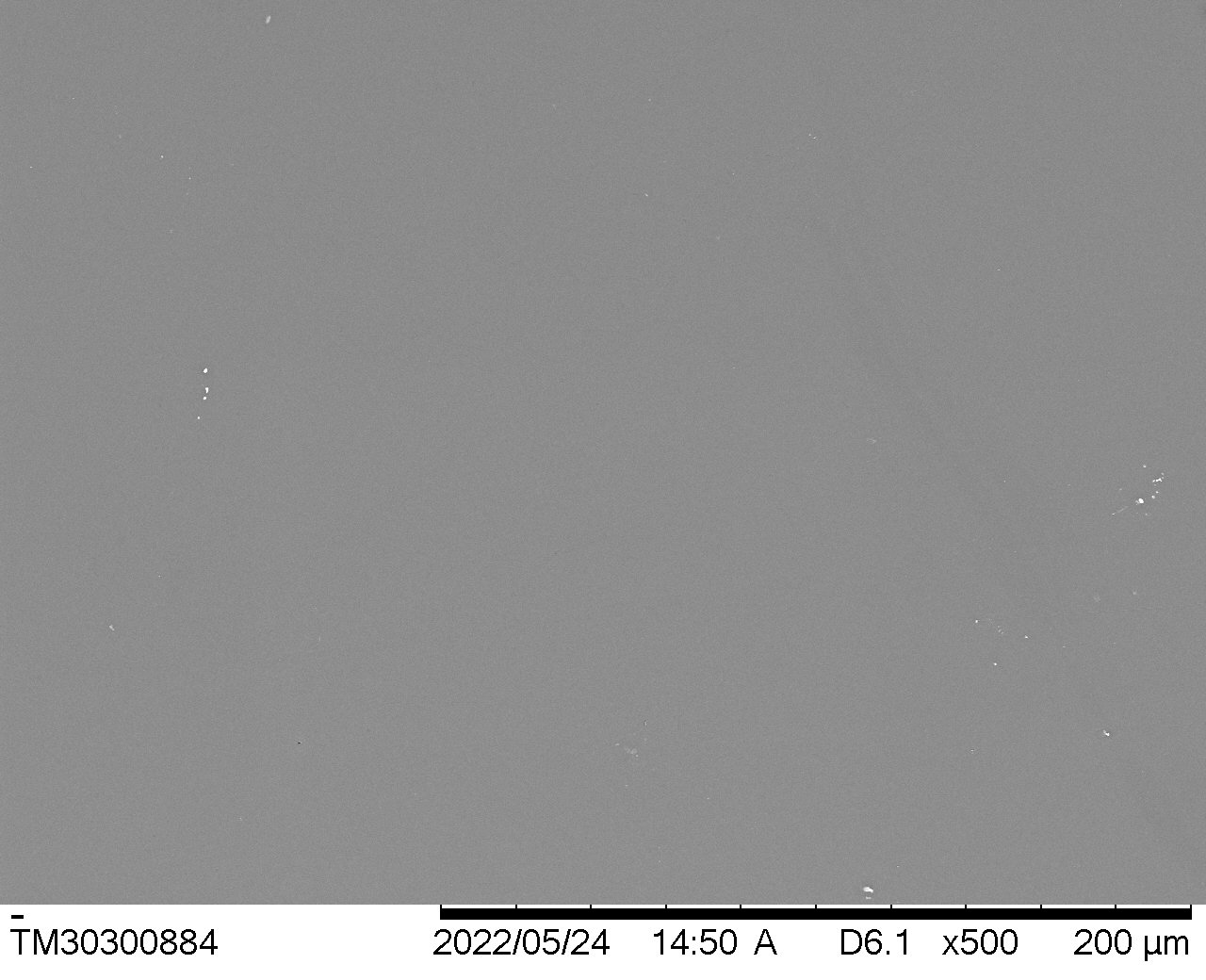

Supplement: Supplementary file 1 [file polymers-17-01414-s001.zip › isx_02_(x500).jpg]

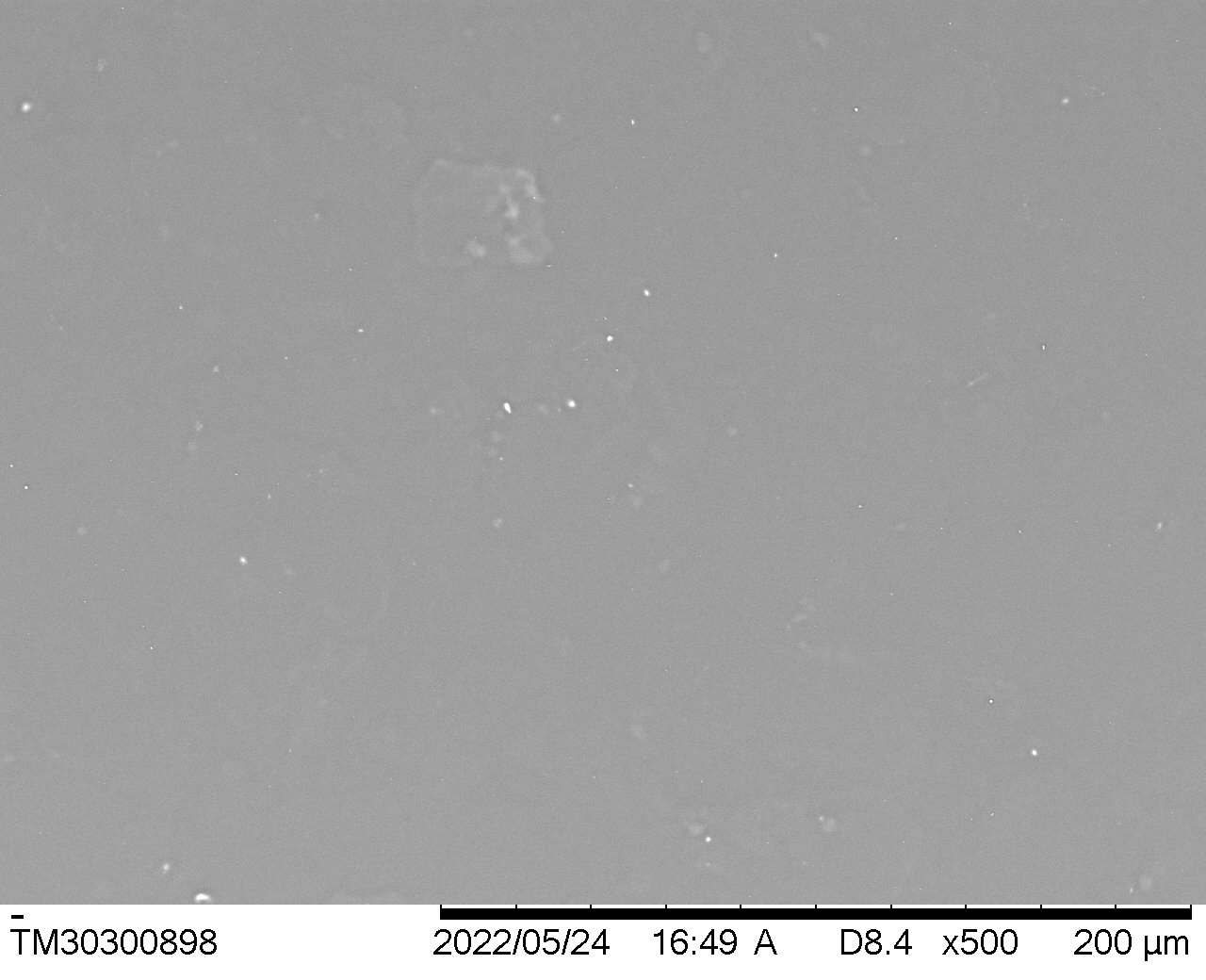

Supplement: Supplementary file 1 [file polymers-17-01414-s001.zip › Pc_250_02_(x500).jpg]

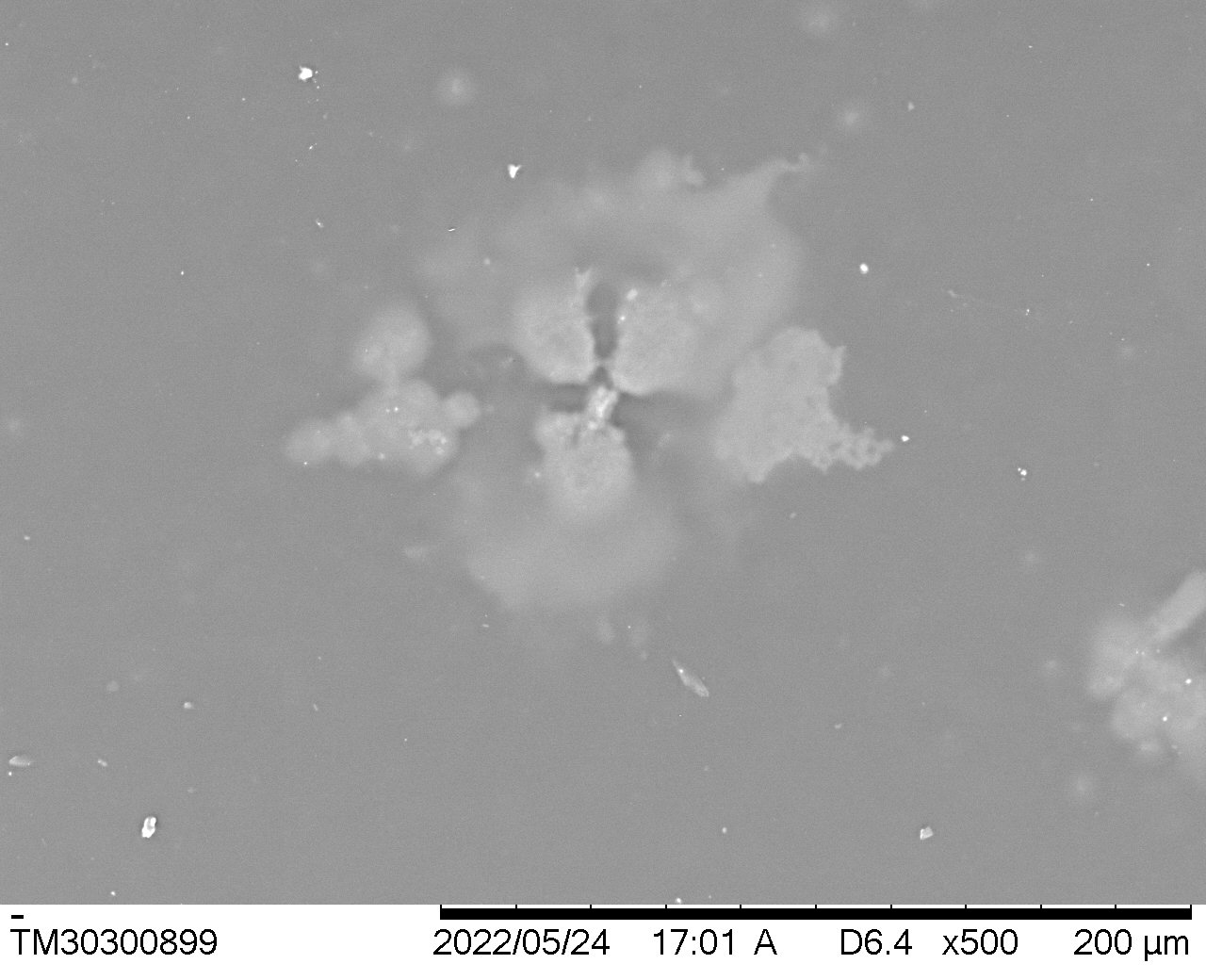

Supplement: Supplementary file 1 [file polymers-17-01414-s001.zip › Pc_720_01_(x500).jpg]

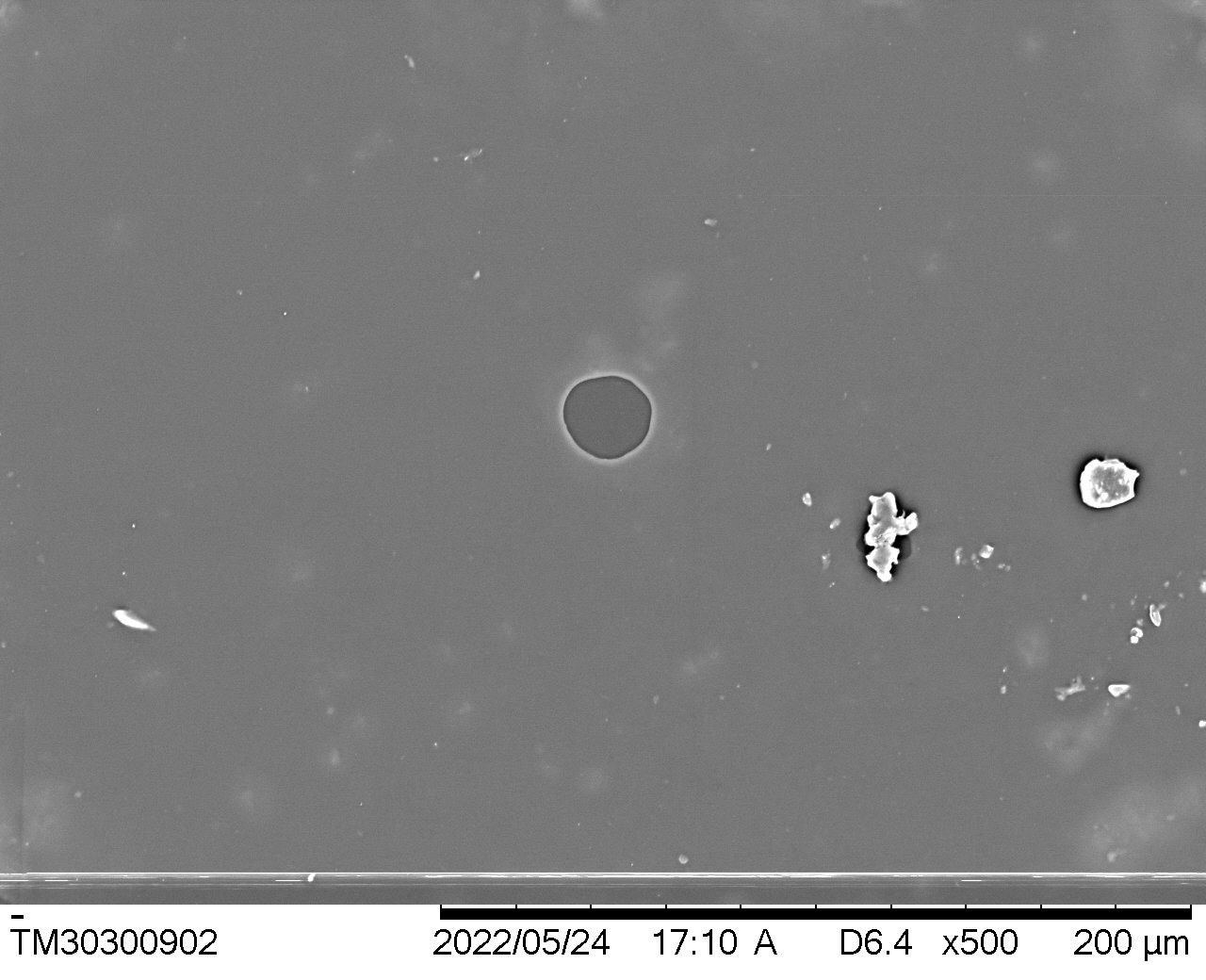

Supplement: Supplementary file 1 [file polymers-17-01414-s001.zip › Pc_720_02_(x500).jpg]

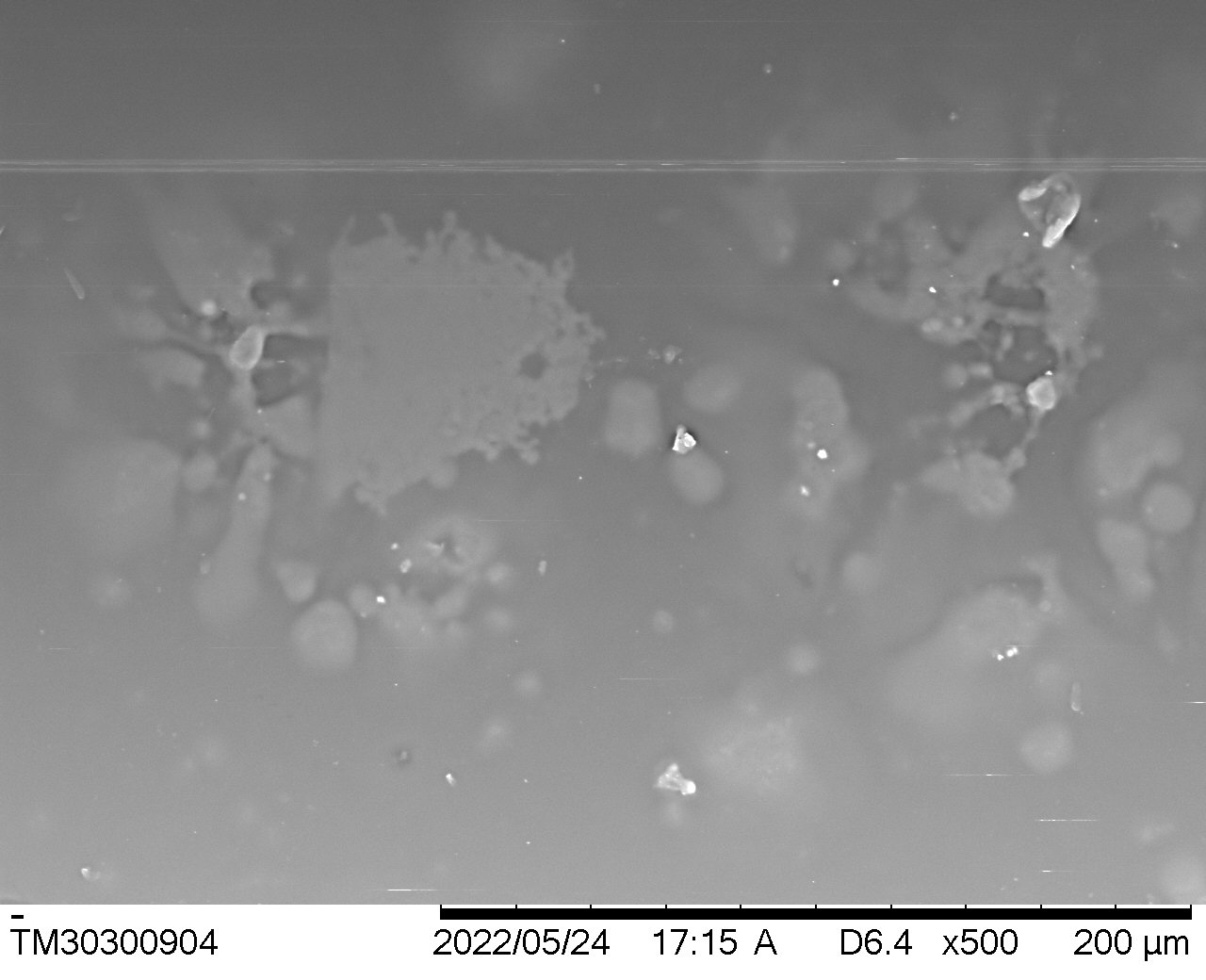

Supplement: Supplementary file 1 [file polymers-17-01414-s001.zip › Pc_720_03_(x500).jpg]

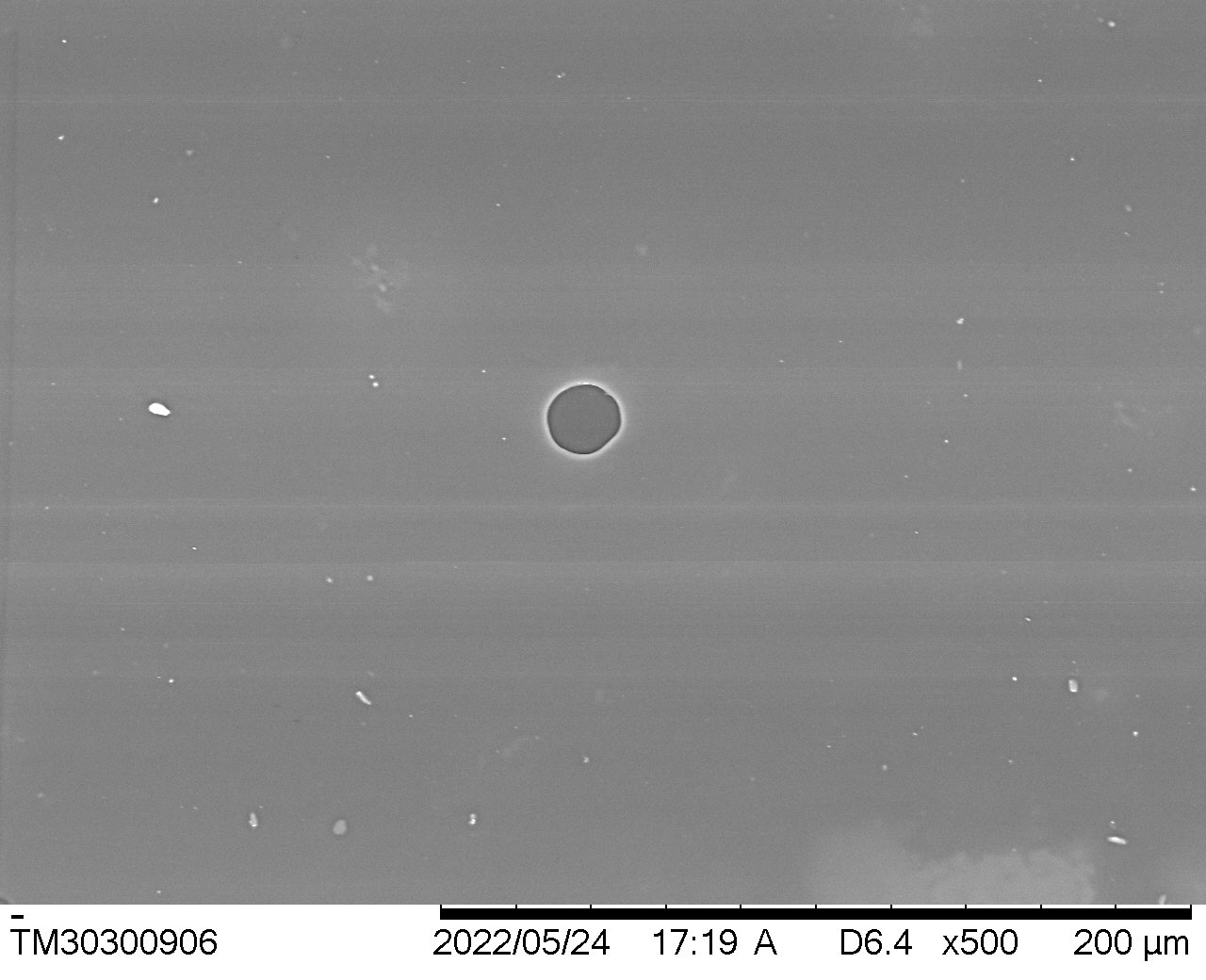

Supplement: Supplementary file 1 [file polymers-17-01414-s001.zip › Pc_720_04_(x500).jpg]

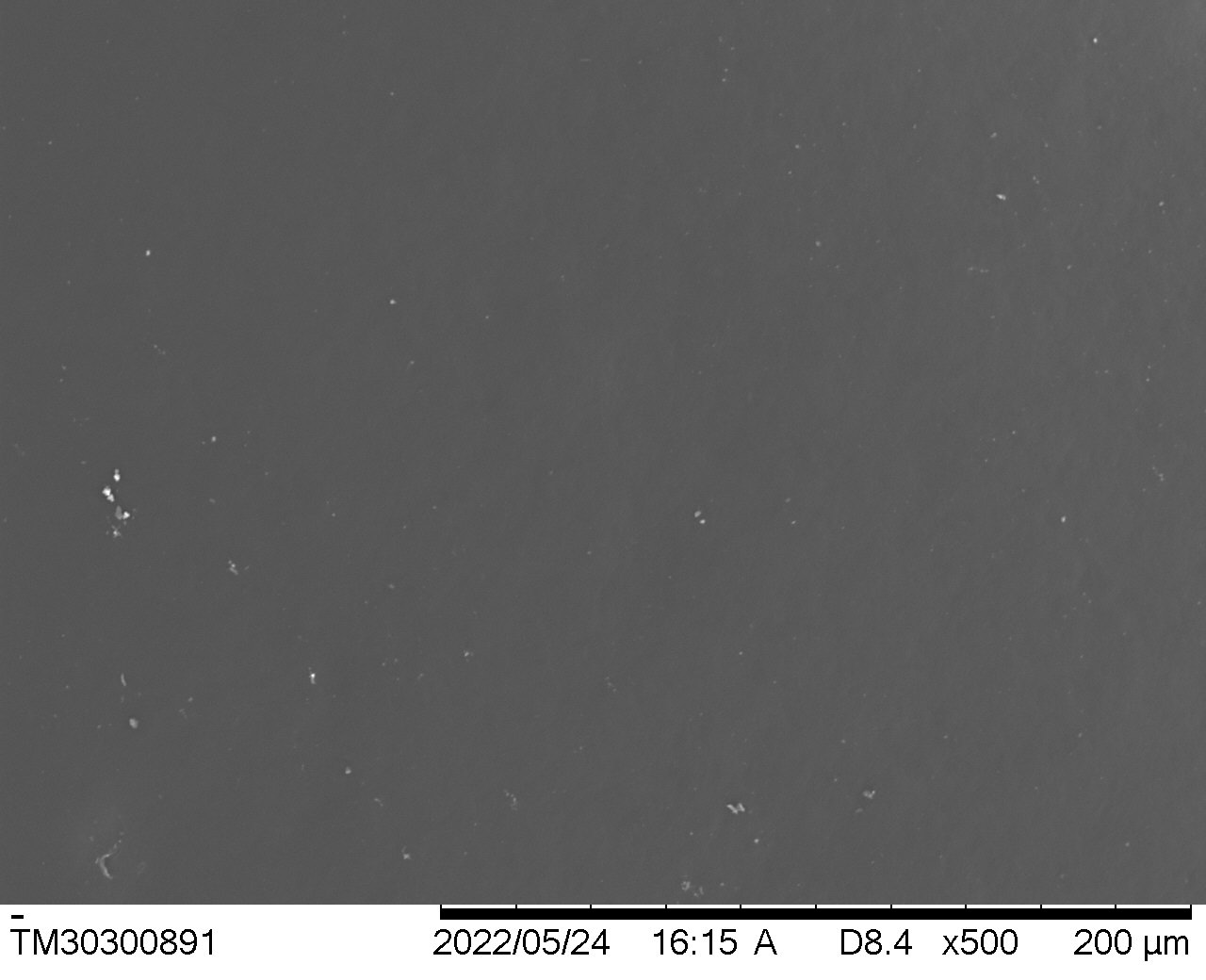

Supplement: Supplementary file 1 [file polymers-17-01414-s001.zip › Pc_isx_01_(x500).jpg]

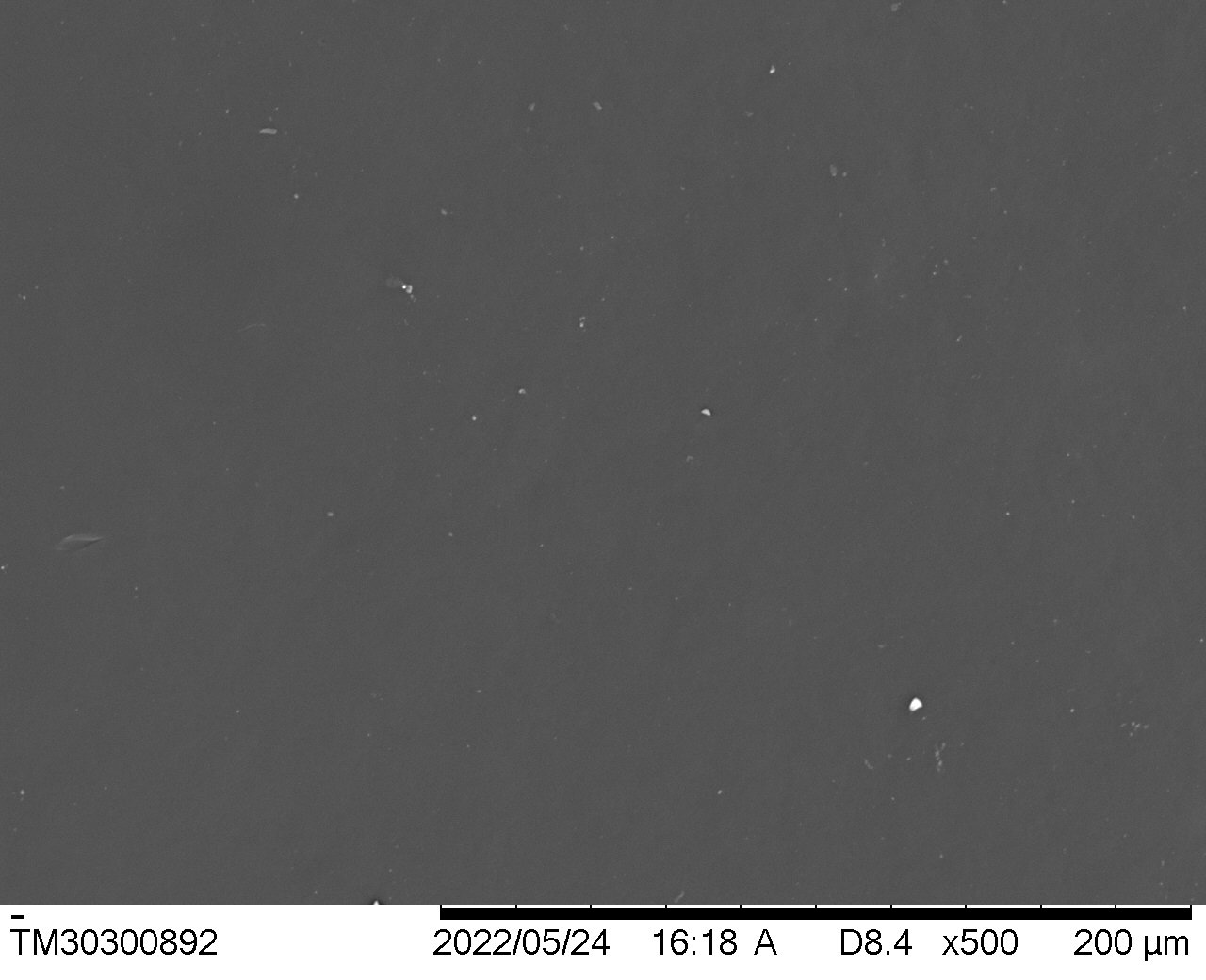

Supplement: Supplementary file 1 [file polymers-17-01414-s001.zip › Pc_isx_02_(x500).jpg]

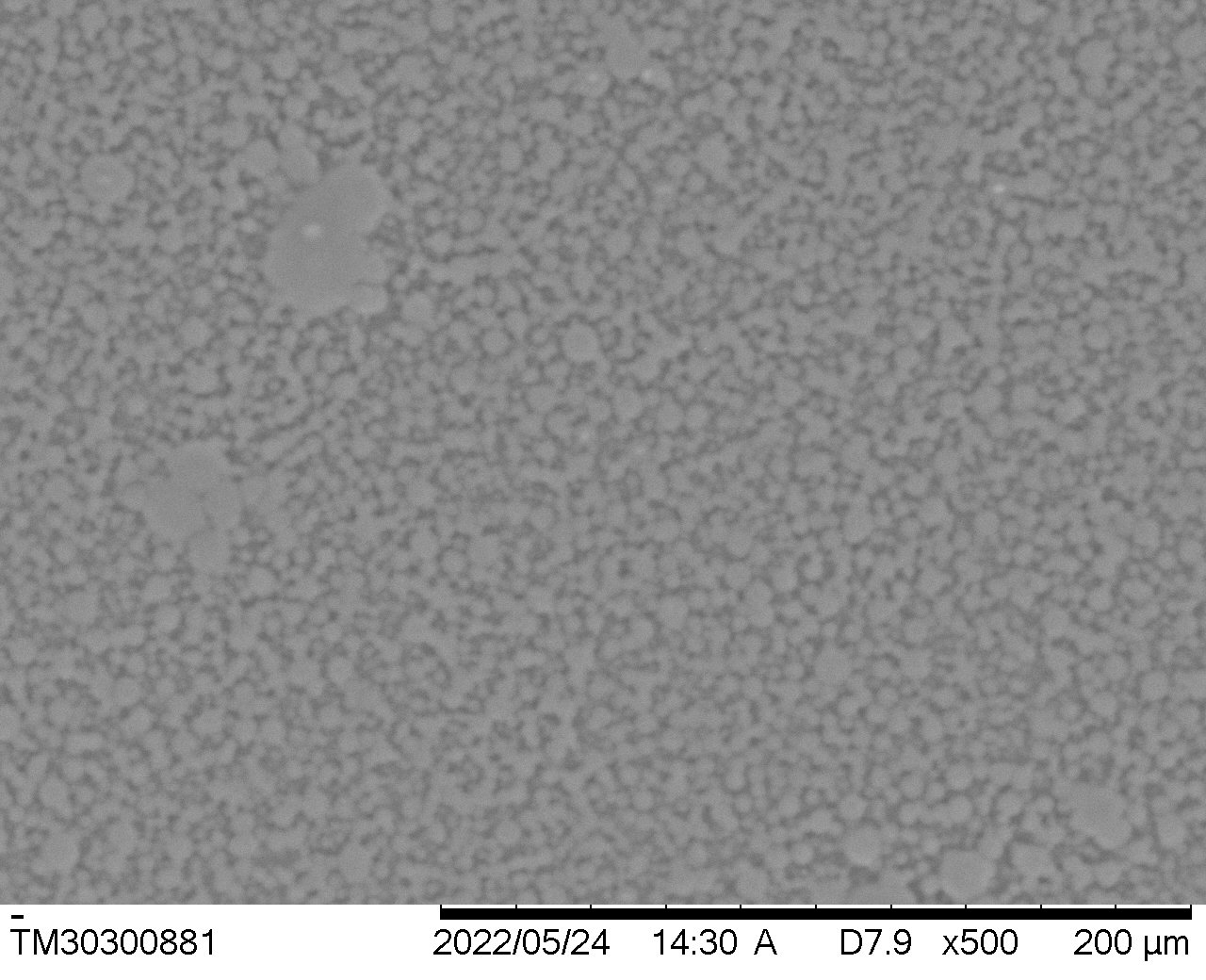

Supplement: Supplementary file 1 [file polymers-17-01414-s001.zip › PMMA_60_01_(x500).jpg]

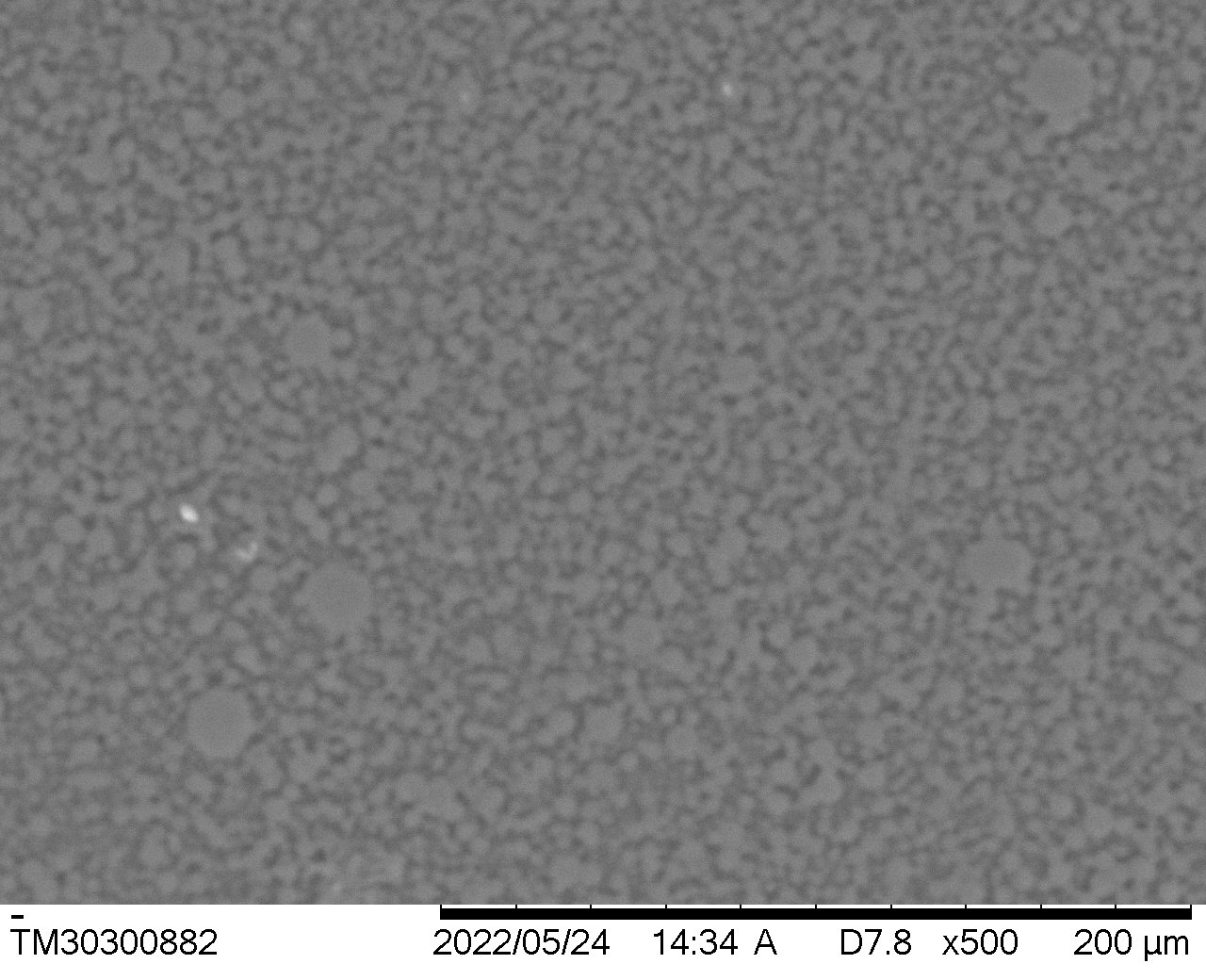

Supplement: Supplementary file 1 [file polymers-17-01414-s001.zip › PMMA_60_02_(x500).jpg]

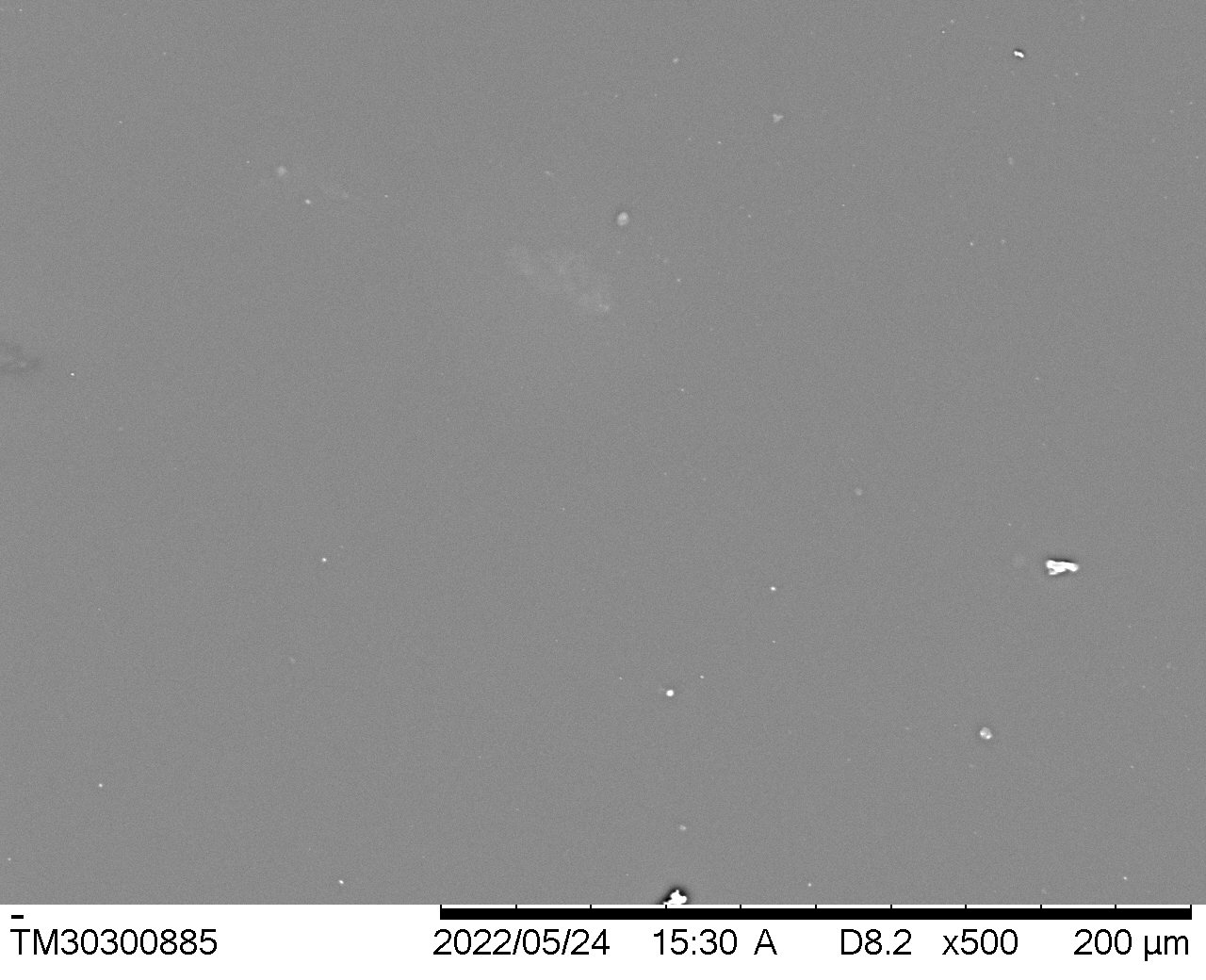

Supplement: Supplementary file 1 [file polymers-17-01414-s001.zip › PMMA_90_01_(x500).jpg]
